# Zika Virus Infection in the Ovary Induces a Continuously Elevated Progesterone Level and Compromises Conception in Interferon Alpha/Beta Receptor-Deficient Mice

**DOI:** 10.1128/JVI.01189-21

**Published:** 2022-01-26

**Authors:** Yingying Zhang, Ziyang Sheng, Na Gao, Na Wu, Peigang Wang, Dongying Fan, Deshan Zhou, Gong Cheng, Jing An

**Affiliations:** a Department of Microbiology, School of Basic Medical Sciences, Capital Medical University, Beijing, China; b Laboratory Animal Center, Capital Medical University, Beijing, China; c Department of Histology and Embryology, School of Basic Medical Sciences, Capital Medical University, Beijing, China; d Tsinghua-Peking Center for Life Sciences, School of Medicine, Tsinghua University, Beijing, China; e Center of Epilepsy, Beijing Institute for Brain Disorders, Beijing, China; University of North Carolina at Chapel Hill

**Keywords:** Zika virus, ovary, estrous cycle, conception, female reproductive health

## Abstract

Zika virus (ZIKV) belongs to mosquito-borne flaviviruses. Unlike other members in the family, ZIKV can be sexually transmitted, and the female genital tracts are susceptible to ZIKV. However, the impact of ZIKV infection on nonpregnant female reproductive health is not understood. In this study, we investigated the effects of ZIKV infection on the ovary by using nonpregnant female interferon α/β receptor-deficient (*Ifnar1^−/−^*) mice. The results showed that the ovary supported ZIKV replication, and the granulosa and theca cells of antral follicles were susceptible. ZIKV replication *in situ* significantly reduced the numbers of antral follicles, aggravated follicular atresia, and disrupted folliculogenesis. Notably, ZIKV replication in the ovary caused disordered ovarian steroidogenesis manifested by decreased expression of key enzymes linked to sex hormone synthesis, including the cytochrome P450 17A1 (CYP17A1) and aromatase (CYP19A1). Further, we observed that ZIKV infection disrupted the estrous cycle and thus prolonged the time to conceive. More importantly, although ZIKV RNA could not be detected at 3 months postinfection, damaged ovarian structure and dysfunction were also observed. Taken together, our study demonstrates that ZIKV infection in nonpregnant female mice cause ovarian damage and dysfunction, even long after ZIKV clearance. These data provide important information to understand the effects of ZIKV infection in female reproductive tissues and basic evidence for further studies.

**IMPORTANCE** Zika virus (ZIKV), a flavivirus, is primarily transmitted by mosquito bites. But it can also be transmitted vertically and sexually. Although ZIKV-associated Guillain-Barré syndrome and microcephaly have drawn great attention, there have been few studies on the potential effects of ZIKV on the genital tract of nonpregnant females. This study investigated the effects of ZIKV on the ovaries in mice. We found that ZIKV replicated in the ovary and the granulosa and theca cells of antral follicles were susceptible. ZIKV replication *in situ* significantly damaged ovarian structure and function and disrupted folliculogenesis. Notably, ZIKV infection further disrupted the estrous cycle and prolonged the time to conceive in mice by causing disordered ovarian steroidogenesis. These effects were observed in both the acute phase and the recovery phase after viral elimination. Overall, the new findings provide important additions to make out the potential adverse impacts of ZIKV on reproductive health in females.

## INTRODUCTION

Zika virus (ZIKV) belongs to the Flaviviridae family and is usually transmitted by mosquito biting. Unlike other flaviviruses, ZIKV can also be transmitted by nonvector routes including sexual and vertical routes. Previously, the effects of ZIKV on male reproductive health and ZIKV-related congenital cerebral malformations in fetuses have attracted much attention (1, 2). However, the consequences of ZIKV on female reproductive health have not been extensively evaluated. A study confirmed that ZIKV RNA was detected in oocytes in a ZIKV-infected patient, suggesting ZIKV infection of the human ovary (3). Moreover, research on nonhuman primates has demonstrated that ZIKV RNA was cleared from the plasma by day 7 after infection, but ZIKV RNA-positive cells persisted in multiple anatomic tissues, including the ovaries (4). Although mouse model studies have demonstrated that ZIKV can replicate in the ovary and cause acute oophoritis (5, 6), several questions urgently need to be clarified. How does ZIKV replication influence ovarian function? What are the long-term effects of ZIKV infection on the ovary? What molecular mechanisms are involved?

Ovarian functions include generating fertilizable oocytes with competence for reproduction (oogenesis) and maintaining endocrine homeostasis and pregnancy establishment (steroidogenesis) (7, 8). Normally, there are many follicles in different stages, including primordial, primary, secondary, mature, and atretic follicles, and the corpora lutea deriving from mature follicles after ovulation in the ovary. The transformation of primary follicles into secondary follicles is usually accompanied by the formation of cavities (also known as antral follicles). An ovarian follicle is composed of numerous granulosa and theca cells (GCs and TCs, respectively) surrounding an oocyte. Cross talk between an oocyte and its circumambient granulosa cells is closely associated with follicular development.

Sex hormones can be synthesized in the growing follicles, especially the antral follicles and the terminally differentiated luteal cell (9–12). Rodents represent a good model to study the effects of abnormal sex hormone fluctuations on the reproductive cycles known as menstrual (in humans) or estrous (in rodents) (13–15). Accordingly, vaginal cytology exhibits a periodic change, and different types of epithelial cells can be observed (16). The normal ovarian cycle is closely related to follicular development and ovulation. More importantly, it provides essential conditions for female reproductive health, particularly conception. Any unfavorable factors may lead to disorder in enzyme synthesis and steroid hormone metabolism, which further damages female reproductive health (17, 18).

Therefore, to uncover the effects of ZIKV on the ovary and the associated mechanism, *Ifnar1^−/−^* mice were inoculated with ZIKV or a related dengue virus (DENV-2), and the infection outcomes of the ovary were monitored. ZIKV infection of the ovaries of interferon α/β and γ receptor-deficient (*Ifnar1^−/−^Ifngr1^−/−^*) mice was also verified. The results revealed that ZIKV but not DENV-2 led to ovarian damage and dysfunction in mice. This study may provide important clues for understanding the impacts of ZIKV infection on female reproductive health.

## RESULTS

### Ovary effectively supports ZIKV replication.

**FIG 1:**
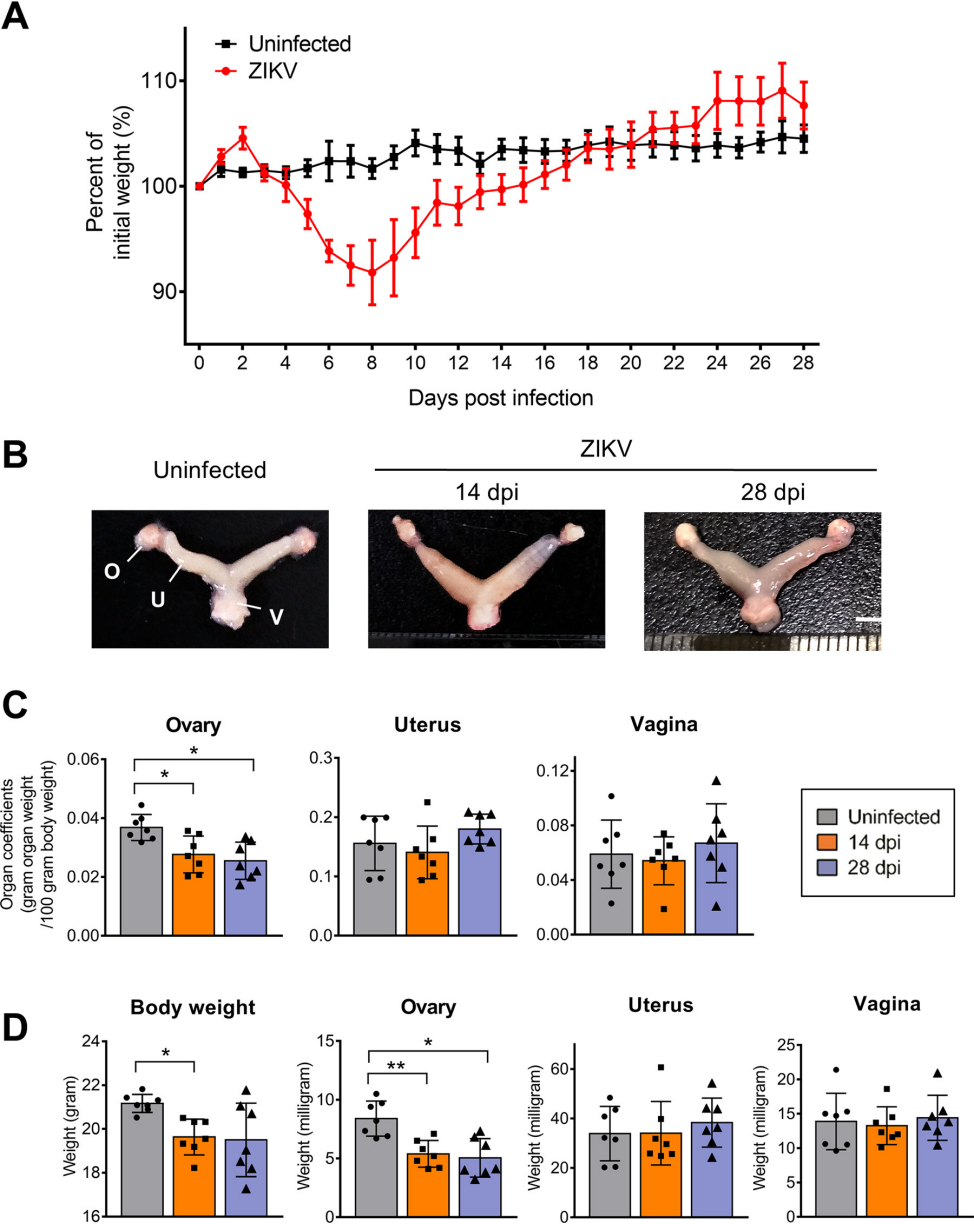
ZIKV infection leads to relative mass loss of the ovary. Adult female *Ifnar1^−/−^* mice 6 to 8 weeks old were subcutaneously inoculated with 10^4^ PFU ZIKV SMGC-1. Ovaries, uteri, and vaginas were collected at 14 and 28 days postinfection (dpi). (A) The body weights of ZIKV-infected mice and uninfected mice were monitored over time (*n* = 5 to 6/group). (B) Representative morphological changes in the female genital organs of uninfected or ZIKV-infected *Ifnar1^−/−^* mice were evaluated at 14 and 28 dpi. O, ovary; U, uterus; V, vagina. Bar = 5 mm. (C) The organ coefficients (g organ weight/100 g body weight) of the ovaries, uterus, and vagina of uninfected mice or ZIKV-infected mice at 14 and 28 dpi were measured to reflect the mass loss of the organs (*n* = 7/group). (D) The changes of body weights, ovarian, uterine, and vaginal weights of ZIKV-infected *Ifnar1^−/−^* mice at 14 and 28 dpi were evaluated. Uninfected mice were considered controls (*n* = 7/group). The data are presented as the means ± standard deviation (SD). Each spot represents an individual mouse. The significance of differences was analyzed by ordinary one-way ANOVA with a Bonferroni's multiple-comparison test (C) and a Tukey’s multiple-comparison test (D). *, *P* < 0.05; **, *P* < 0.01.

**FIG 2:**
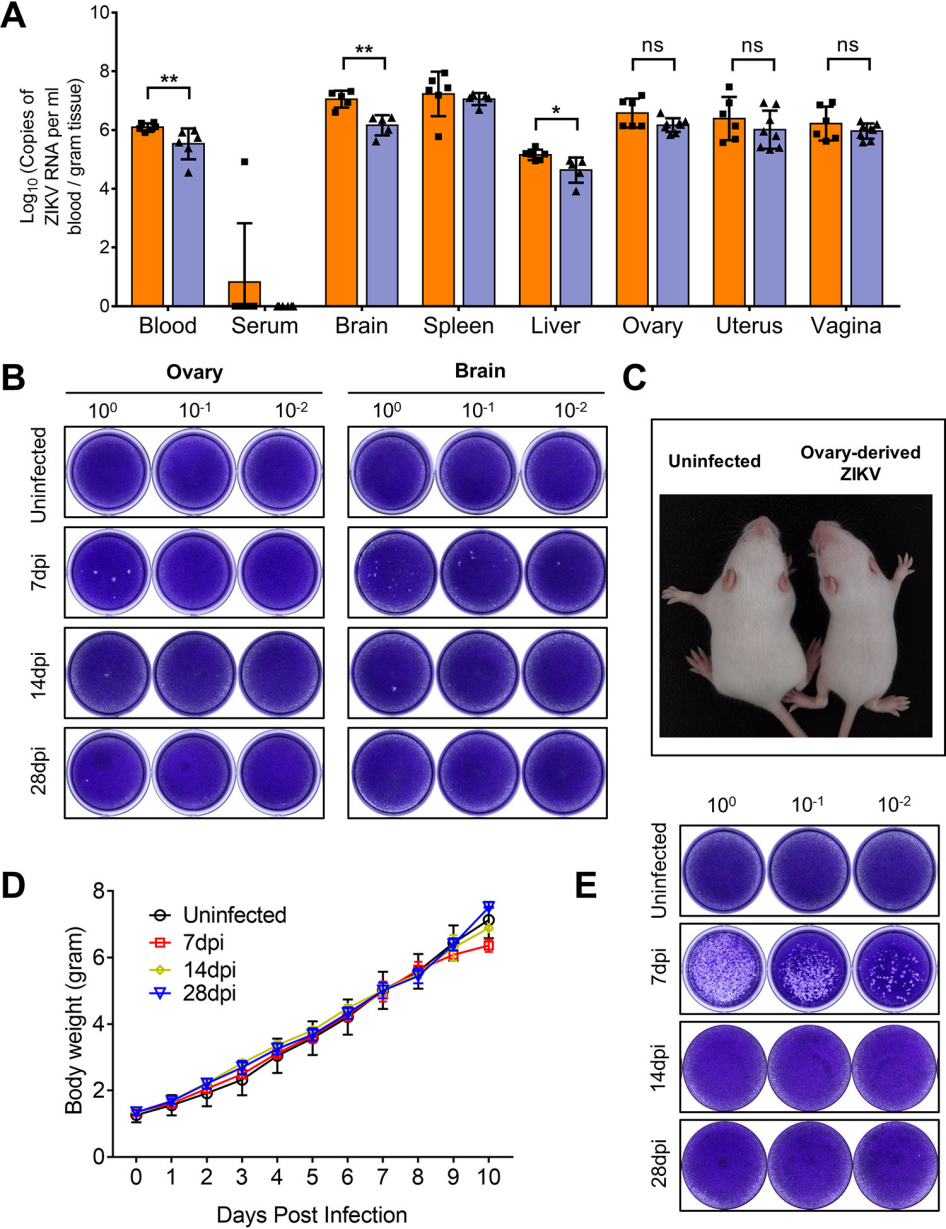
ZIKV can replicate in the ovary. Adult female *Ifnar1^−/−^* mice 6 to 8 weeks old were subcutaneously inoculated with 10^4^ PFU ZIKV SMGC-1. (A) The viral RNA loads in the blood, serum, major organs, ovaries, uteri, and vaginas of ZIKV-infected *Ifnar1^−/−^* mice at 14 and 28 dpi were analyzed by RT-qPCR (*n* = 5 to 8/group). (B) Infectious ZIKV particles in the ovary and brain (positive control) of uninfected or ZIKV-infected mice at 7, 14, and 28 dpi were determined by plaque assay. The ovaries of five individual mice were pooled for each time point in both groups. (C) One-day-old BALB/c suckling mice were intracerebrally inoculated with ovarian homogenate. Representative image of growth delay in a suckling mouse inoculated with ovarian homogenate derived from the ovaries of ZIKV-infected *Ifnar1^−/−^* mice at 7 dpi. A littermate was inoculated with PBS as a control. (D) The body weights of the suckling mice inoculated with the ovary-derived ZIKV or with PBS were monitored over time (*n* = 6/group). (E) The brains of the suckling mice were collected 8 to 10 days after inoculation and were pooled. Infectious ZKIV particles in the brains of suckling mice were determined by plaque assay. The data are presented as the means ± SD. Each spot represents an individual mouse. The significance of the differences was analyzed by a Mann-Whitney test (A). *, *P* < 0.05; **, *P* < 0.01. ns, no significant difference.

To characterize ZIKV infection in female reproductive organs, nonpregnant female *Ifnar1^−/−^* mice were subcutaneously inoculated with 10^4^ PFU ZIKV. The ovaries, uteri, vaginas, serum, blood, and other organs were collected at 14 and 28 days postinfection (dpi). After ZIKV infection, the mice started losing weight at 4 dpi, with the lowest body weights observed between 7 and 9 dpi, and then gradually returned to normal (Fig. 1A). The dissected ovaries displayed obvious petechia and shrinkage (Fig. 1B). Organ coefficients (the ratio of wet organ weight to body weight) were measured to reflect the degree of lesion (19). In uninfected mice, the ovarian organ coefficient was 0.037 ± 0.004. The coefficient dropped to 0.028 ± 0.006 at 14 dpi and 0.025 ± 0.006 at 28 dpi (Fig. 1C), although the body weights in the infected group were lower than in the uninfected groups (Fig. 1D). In contrast, no significant changes in organ coefficients were observed for the uterus or vagina. Viral RNA was detected in the female reproductive organs, other major organs, serum, and blood. ZIKV RNA levels ranging from 5 to 8 (log_10_) copies per gram or mL were detected in female reproductive tract, the brain, the spleen, and the blood at 14 dpi (Fig. 2A). However, as the infection progressed, the viral load in the blood obviously decreased from 6.10 ± 0.12 (log_10_) copies/mL at 14 dpi to 5.53 ± 0.48 (log_10_) copies/mL at 28 dpi, but those in the ovaries, uterus, vagina, and spleen were maintained at high levels (Fig. 2A), indicating that those organs effectively supported ZIKV replication. Notably, in contrast to the blood samples, viral RNA was detectable in only one serum sample at 14 dpi, indicating that ZIKV RNA could persist in blood longer than in serum (20, 21).

To determine whether infectious ZIKV could replicate in the ovary, the ovaries and brains at 7, 14, and 28 dpi were collected for plaque assays; the brain, an important target organ of ZIKV, was used for positive control (22, 23). The results showed that at 7 dpi, infectious ZIKV particles could be detected in the ovaries and brain. However, at 14 and 28 dpi, few viral plaques were observed (Fig. 2B). To further confirm above results, 1-day-old BALB/c suckling mice were intracerebrally inoculated with the ovarian homogenates derived from *Ifnar1^−/−^* mice infected with ZIKV at 7, 14, and 28 dpi, and the body weights were monitored daily. After inoculation with the ovarian homogenates derived from *Ifnar1^−/−^* mice infected with ZIKV at 7 dpi for 9 days, the suckling mice were manifested by slow movement and growth delay (Fig. 2C and D), and infectious ZIKV particles at 4.52 (log 10) PFU/g tissues were detected in the brain of the suckling mice (Fig. 2E). In contrast, the suckling mice inoculated with ovarian homogenates from *Ifnar1^−/−^* mice infected with ZIKV at 14 and 28 dpi did not develop signs of ZIKV infection (Fig. 2D and E). These data demonstrated that infectious ZIKV particles could be isolated in the ovary in the acute phase of infection, which was consistent with the previous study (24).

**FIG 3:**
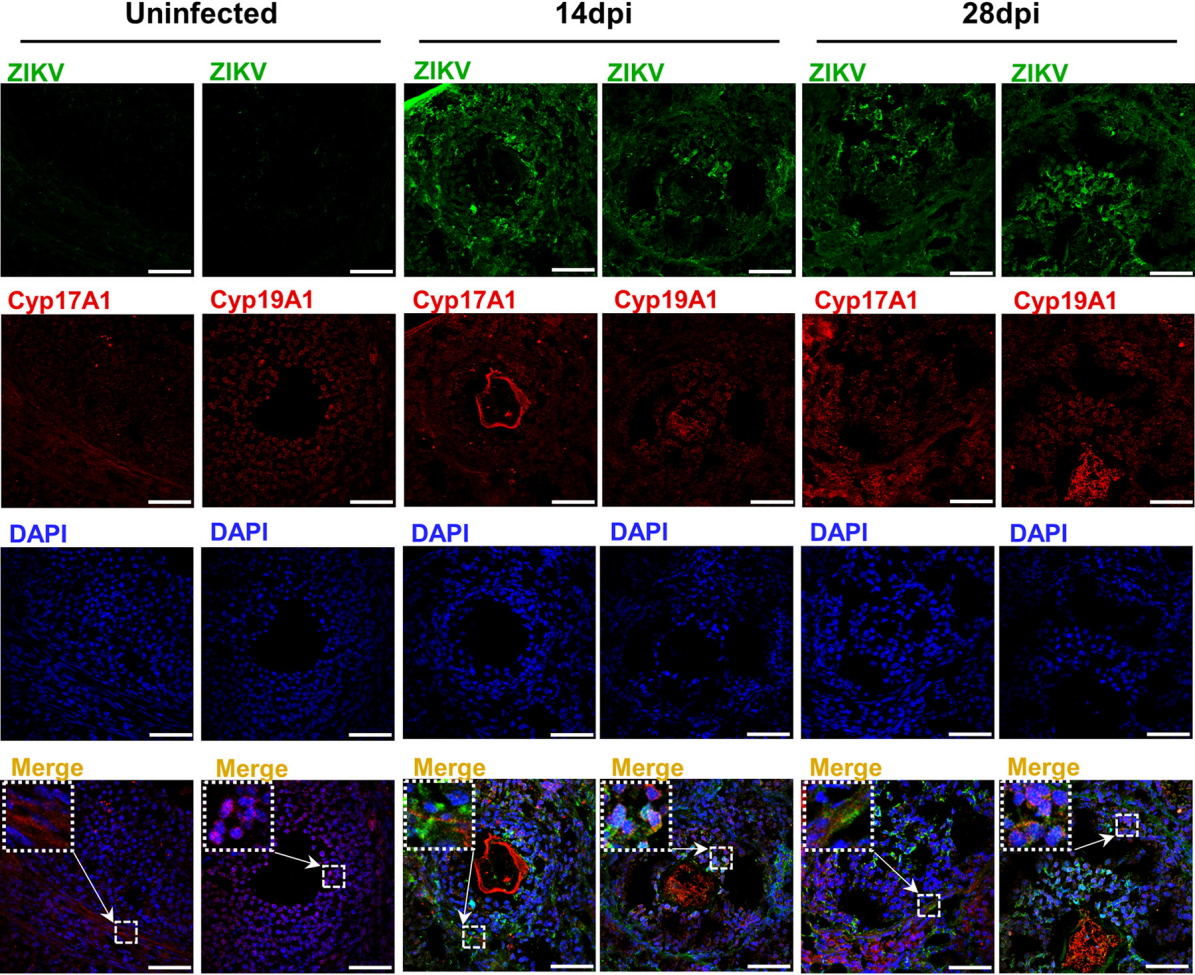
Theca cells and granulosa cells are principal target cells for ZIKV replication in the ovary. Adult female *Ifnar1^−/−^* mice 6 to 8 weeks old were subcutaneously inoculated with 10^4^ PFU ZIKV SMGC-1. The ovaries were collected at 14 and 28 dpi. The distributions of ZIKV in the ovaries at 14 and 28 dpi were detected by IFA. Uninfected ovaries served as controls. ZIKV-positive cells are shown in green, Cyp17A1 staining for theca cells or Cyp19A1 staining for granulosa cells is shown in red, and DAPI staining of nuclei is shown in blue. Cyp17A1, cytochrome P450 17A1; Cyp19A1, aromatase. Bar = 50 μm.

Next, we focused on the susceptible cells of ZIKV in the ovary. As revealed by an immunofluorescence staining assay (IFA), viral antigens were distributed around follicles in different developmental stages and scattered in the inner parts of antral follicles at 14 and 28 dpi (Fig. 3). Moreover, the viral antigens surrounding the follicles colocalized with CYP17A1, the marker of theca cells, and those scattered within follicles colocalized with CYP19A1, the marker of granulosa cells, indicating that theca cells and granulosa cells were principal target cells for ZIKV in the ovary.

### ZIKV infection significantly disrupts antral follicles in the ovary.

**FIG 4:**
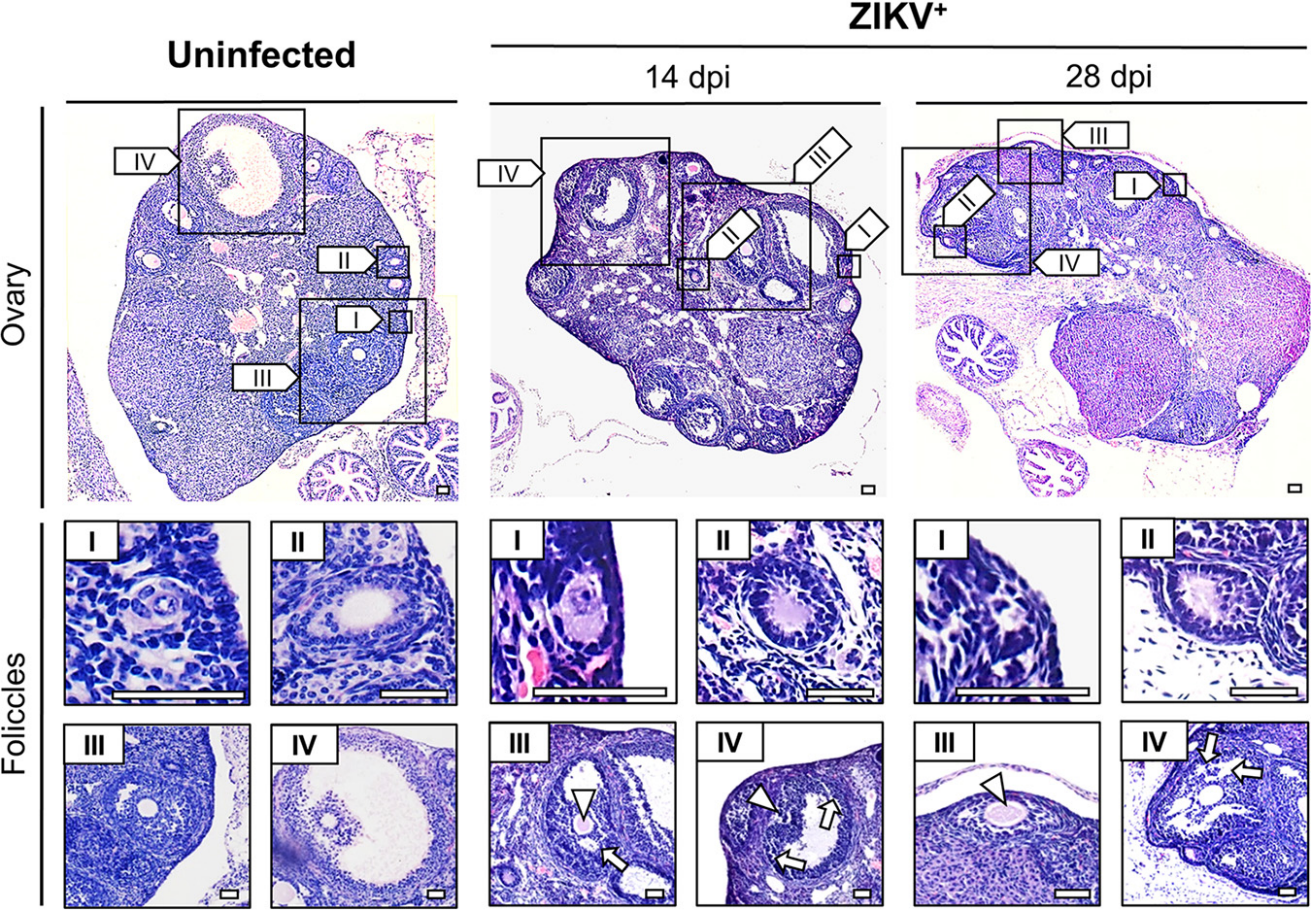
ZIKV infection damages ovarian structure. Adult female *Ifnar1^−/−^* mice 6 to 8 weeks old were subcutaneously inoculated with 10^4^ PFU ZIKV SMGC-1. The ovaries were collected at 14 and 28 dpi. Histopathological changes in the ovaries of uninfected and ZIKV-infected *Ifnar1^−/−^* mice were analyzed at 14 and 28 dpi by HE staining. Bar = 50 μm. Oocyte shrinkage (arrowheads) and clefts in loosely arranged granulosa cell layers (arrows) are indicated. I, primordial follicles; II, primary follicles; III, secondary follicle; IV, mature follicle.

**FIG 5:**
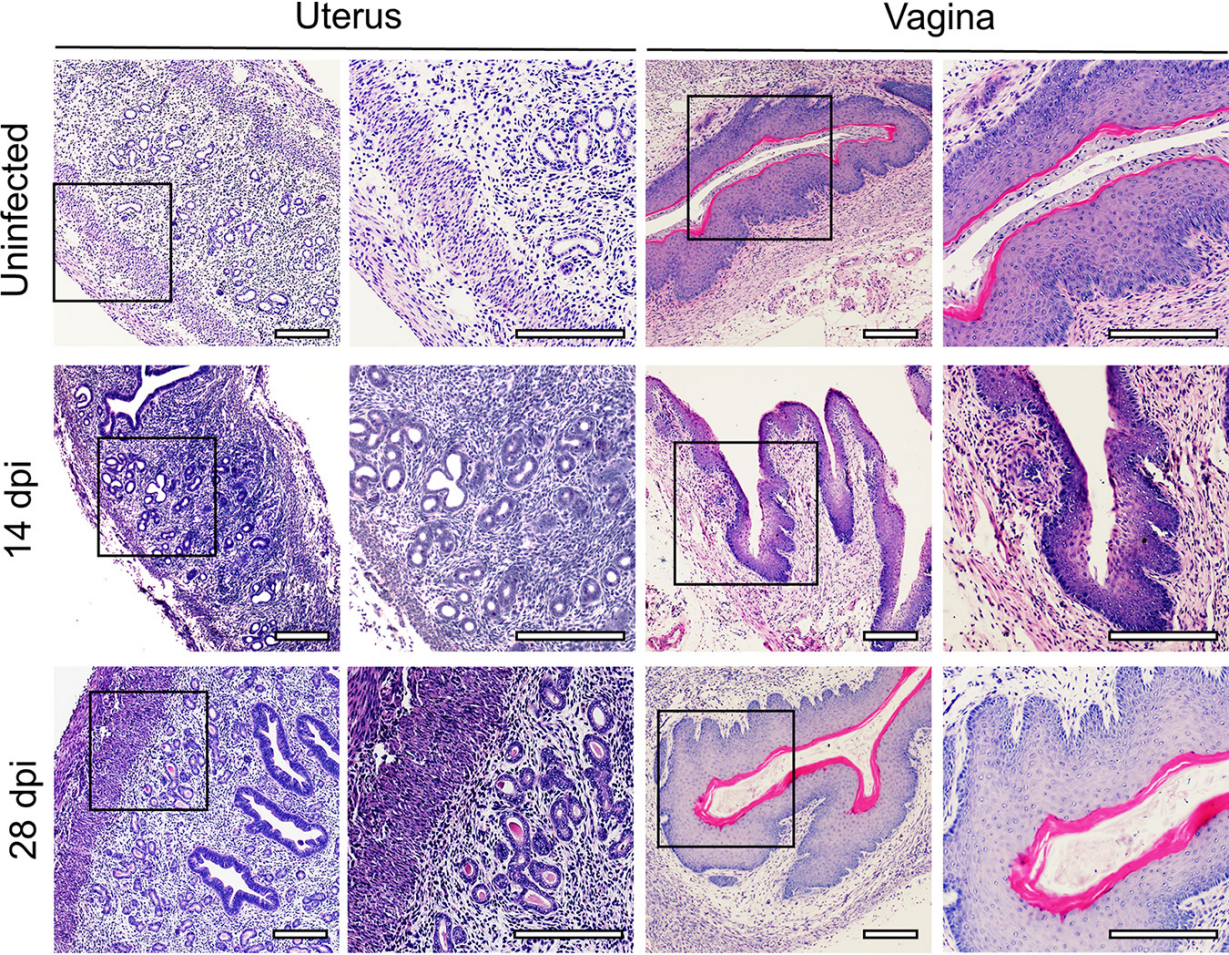
Histopathology of the uterus and vagina after ZIKV infection. Adult female *Ifnar1^−/−^* mice 6 to 8 weeks old were subcutaneously inoculated with 10^4^ PFU ZIKV SMGC-1. ZIKV-infected uteri and vaginas were collected at 14 and 28 dpi. Histopathological changes were analyzed by HE staining. Uninfected uteri and vaginas were considered controls. Bar = 200 μm.

The impacts of ZIKV on the histomorphology of the ovaries were investigated by hematoxylin and eosin (HE) staining. In uninfected ovaries, follicles in different developmental stages were noticeable, and the boundaries between follicles and interstitial tissues were well defined. In contrast, interstitial tissue in ZIKV-infected ovaries showed obviously loose and disordered morphology at 14 and 28 dpi (Fig. 4), while limited histomorphological changes were observed in the uterus and vagina (Fig. 5).

**FIG 6:**
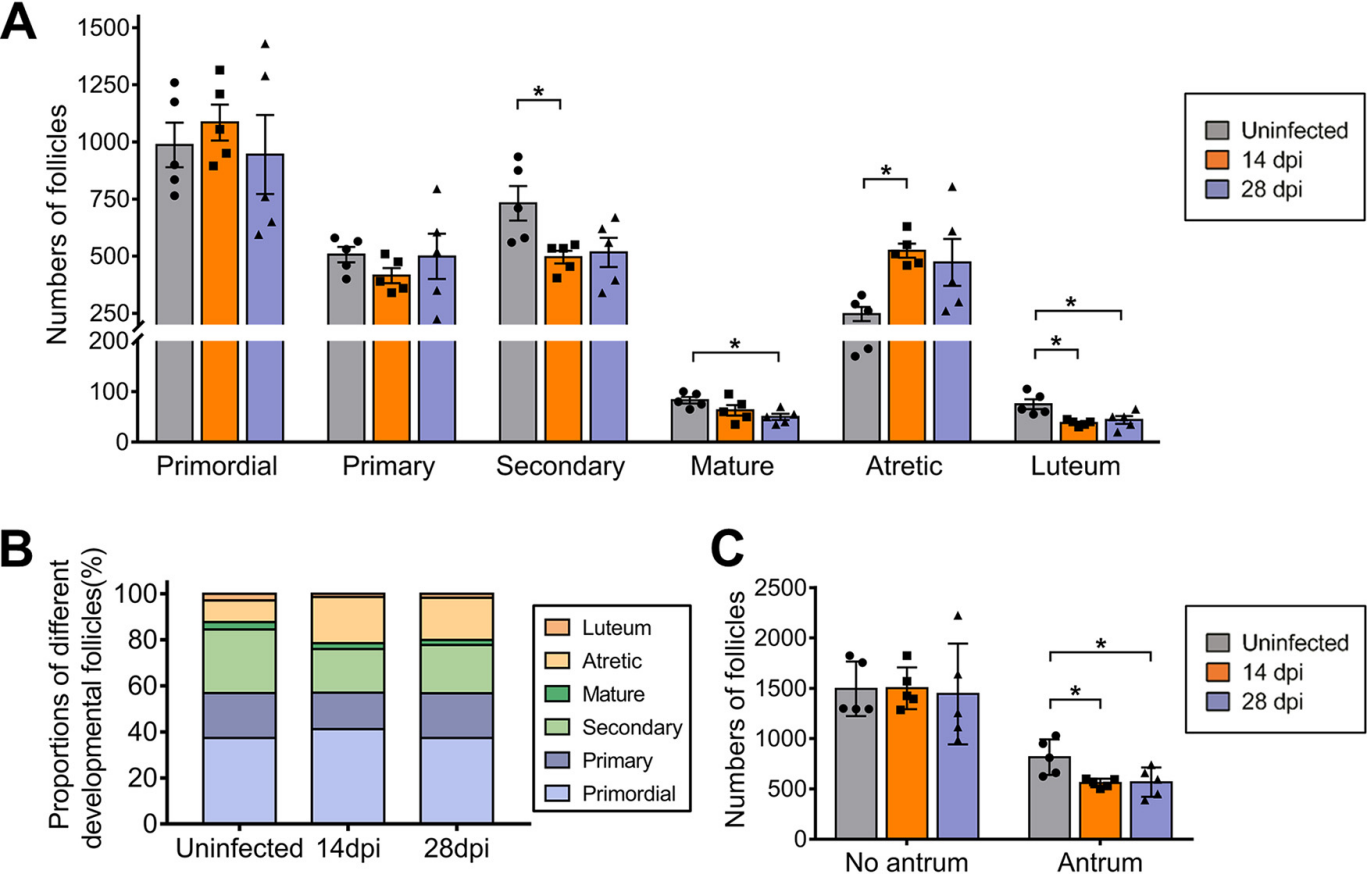
ZIKV infection reduces antral follicle numbers. Adult female *Ifnar1^−/−^* mice 6 to 8 weeks old were subcutaneously inoculated with 10^4^ PFU ZIKV SMGC-1. The ovaries were collected at 14 and 28 dpi. (A, B) The counts (A) and proportions (B) of follicles in different developmental stages and the corpus luteum were determined. (C) The counts of follicles with no antrum or an antrum were calculated (*n* = 5/group). The data are presented as the means ± SEM (A) or the mean ± SD (C). Each spot represents an individual mouse. The significance of differences was analyzed by ordinary one-way ANOVA with a Bonferroni's multiple-comparison test (A, C). *, *P* < 0.05.

The most significant changes observed after ZIKV infection were in the quantities of antral follicles and atretic follicles (Fig. 6). In uninfected ovaries, there were many antral follicles with a substantial antrum and limited numbers of atretic follicles. However, at 14 dpi, the number of antral follicles decreased, which was accompanied by oocyte shrinkage and a loose, thin granulosa cell layer (Fig. 4 and 6C). At 28 dpi, the above changes were more obvious, including significantly decreased numbers of mature follicles and corpora lutea, accompanied by increased atretic follicle numbers (Fig. 6A and B). Notably, ZIKV significantly decreased antral follicle numbers rather than the numbers of follicles without an antrum (Fig. 6C).

### The decrease in antral follicle numbers is attributed to folliculogenesis stunting and granulosa cell apoptosis.

**FIG 7:**
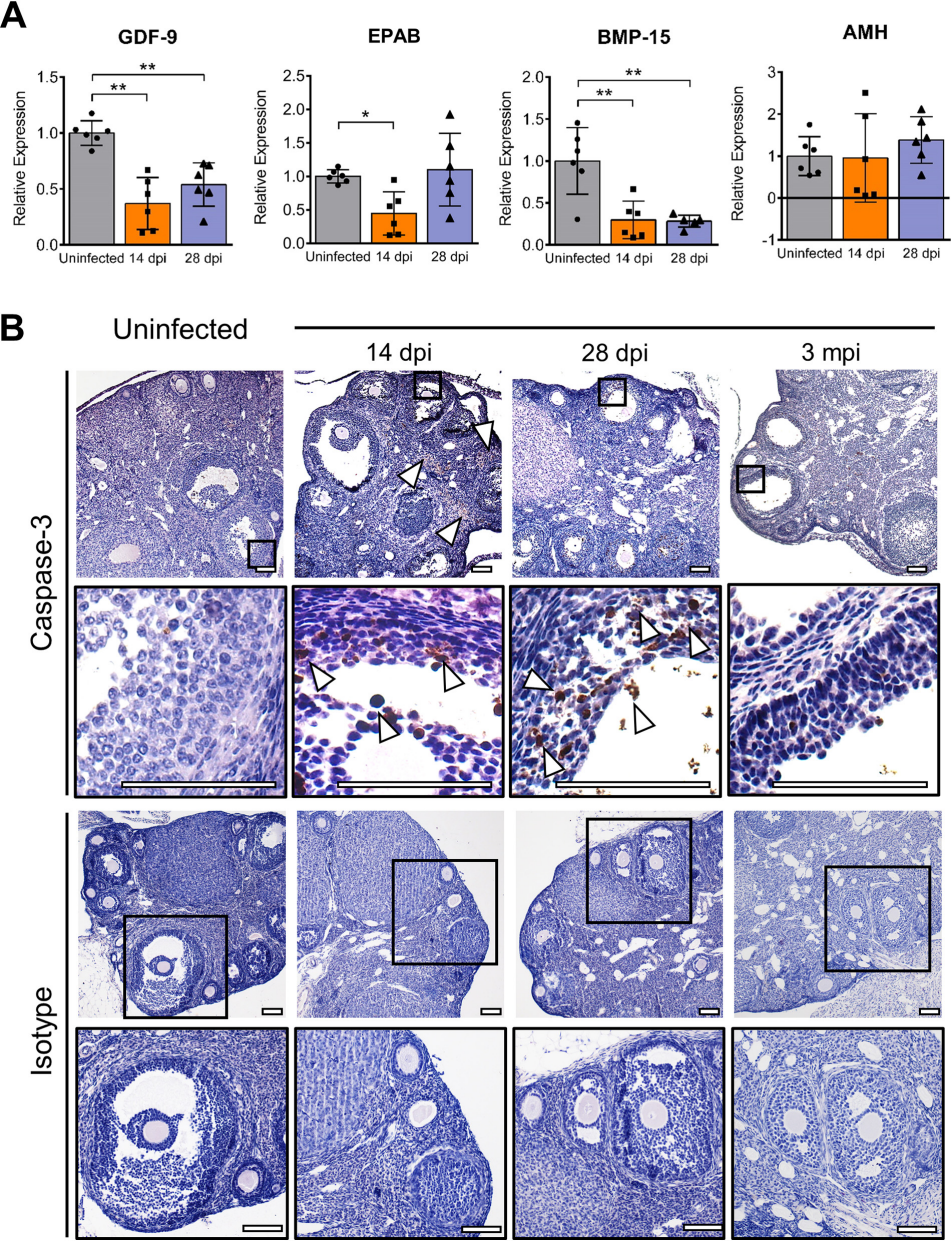
The detection of folliculogenesis-related genes and apoptosis in the ovary. Adult female *Ifnar1^−/−^* mice 6 to 8 weeks old were subcutaneously inoculated with 10^4^ PFU ZIKV SMGC-1. The ovaries were collected at 14 dpi and 28 dpi. (A) The changes in the relative mRNA levels of key mediators of folliculogenesis were determined at 14 and 28 dpi by qRT-PCR. GDF-9, growth differentiation factor-9; EPAB, embryonic poly(A)-binding protein; BMP-15, bone morphogenic protein-15; and AMH, anti-Müllerian hormone (*n* = 6/group). (B) Cleaved caspase-3 expression in ovarian antral follicles was assessed after ZIKV infection. ZIKV-infected ovaries collected at 14 and 28 dpi and 3 mpi were analyzed by IHC staining. Uninfected ovaries were considered controls. Arrowheads indicate positive cleaved caspase-3 staining. Bar = 50 μm. A rabbit IgG isotype control antibody was used to assess the specificity of a rabbit anti-cleaved caspase-3 antibody. Bar = 100 μm. The data are presented as the means ± SD. Each spot represents an individual mouse. The significance of differences was analyzed by ordinary one-way ANOVA with a Tukey’s multiple-comparison test (A). *, *P* < 0.05. **, *P* < 0.01.

To explore the mechanism underlying the decreased numbers of antral follicles, we detected the mRNA levels of main ovarian factors, including GDF-9, BMP-15, EPAB, and AMH, which are exclusively or predominantly expressed by oocytes or granulosa cells and are required for folliculogenesis from primary follicles to secondary follicles or more mature stages (25–30). The results showed that the levels of GDF-9, BMP-15, and EPAB, as promotive factors in folliculogenesis, were all markedly downregulated and reached the lowest levels at 14 dpi (Fig. 7A). At 28 dpi, the GDF-9 and BMP-15 levels in the infected group remained lower than those in the uninfected group, while EPAB levels returned to normal. However, the level of AMH, the major inhibitory factor in folliculogenesis, remained unaltered during infection (Fig. 7A). The imbalanced expression of the above-mentioned genes involved in regulating folliculogenesis was strongly associated with decreased numbers of antral follicles after ZIKV infection.

Cleaved caspase-3 was detected to assess the activation of apoptosis by immunohistochemistry (IHC) staining (31, 32). Compared with the uninfected group, apoptosis of granulosa cells located in the inner surface and cumulus cells in antral follicles and positive staining increased progressively over time in the ZIKV-infected group. Apoptotic cells in interstitial tissues were also observed (Fig. 7B). These changes were not observed when the sections were incubated with an isotype control antibody. Additionally, disordered arrangement of granulosa cells was observed in almost all antral follicles after infection. These results indicated the significantly disrupted architecture of antral follicles, which might accelerate follicular atresia. Except for the decreased numbers of antral follicles in the ZIKV infection group, there were no significant differences in the numbers of primordial follicles or primary follicles among all groups. Taken together, these results suggested that ZIKV infection mainly damaged antral follicles and that folliculogenesis stunting and granulosa cell apoptosis together could lead to decreased numbers of antral follicles.

**FIG 8:**
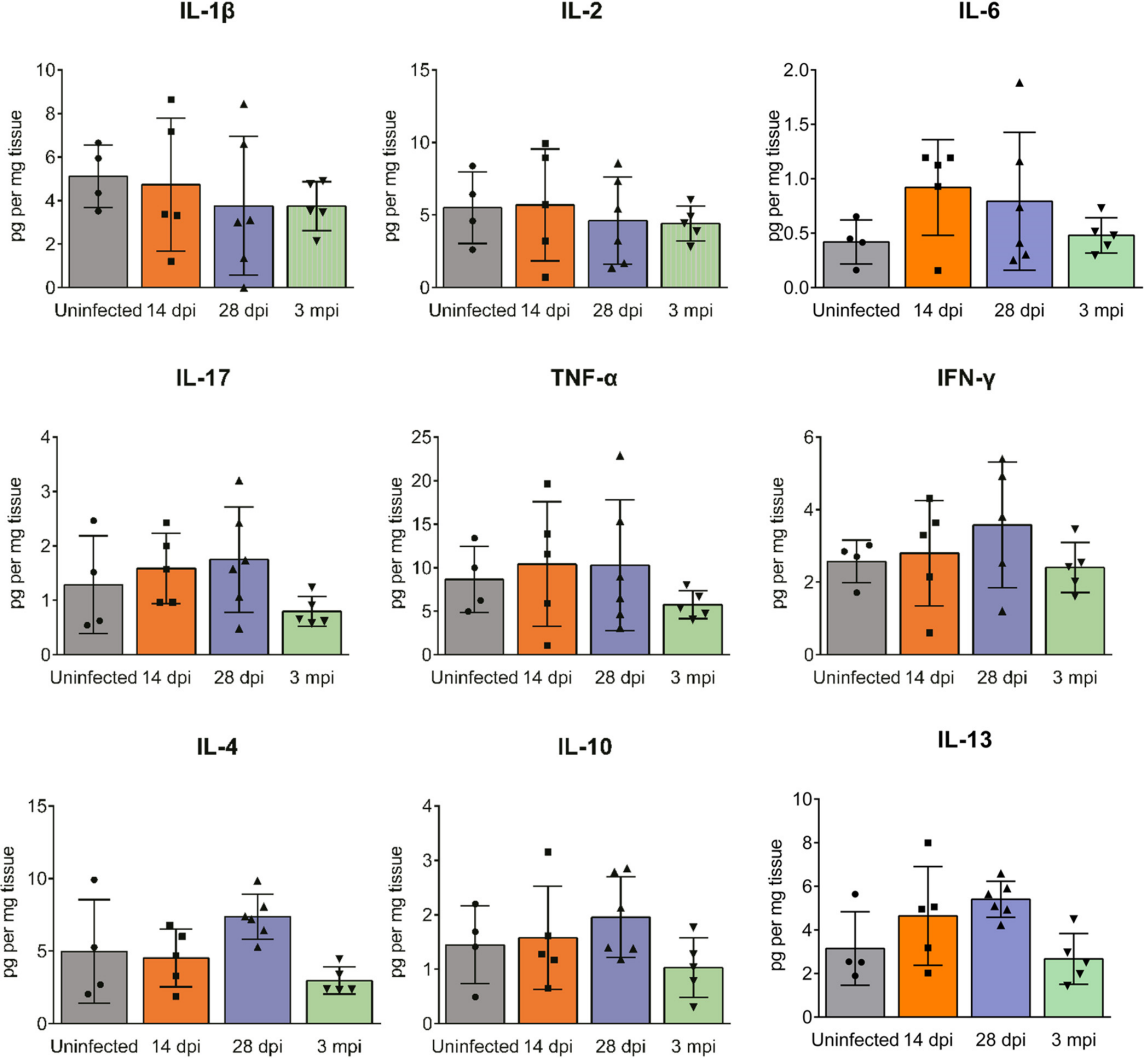
The changes in ovarian cytokines after ZIKV infection. Adult female *Ifnar1^−/−^* mice 6 to 8 weeks old were subcutaneously inoculated with 10^4^ PFU ZIKV SMGC-1. ZIKV-infected ovaries were collected at 14 and 28 dpi and 3 mpi. ZIKV-infected or uninfected ovaries were subjected to cytokine analysis by Bio-Plex multiplex immunoassays. The data are presented as the means ± SD. Each spot represents an individual mouse (*n* = 4 in the uninfected group; *n* = 5 to 6 in the infected groups). IL, interleukin.

Additionally, ovarian cytokine and chemokine profiles were detected with Bio-Plex multiplex immunoassays. The results revealed that cytokine levels did not change much after ZIKV infection (Fig. 8), indicating limited inflammation in the ovaries.

### ZIKV infection causes disordered ovarian steroidogenesis.

**FIG 9:**
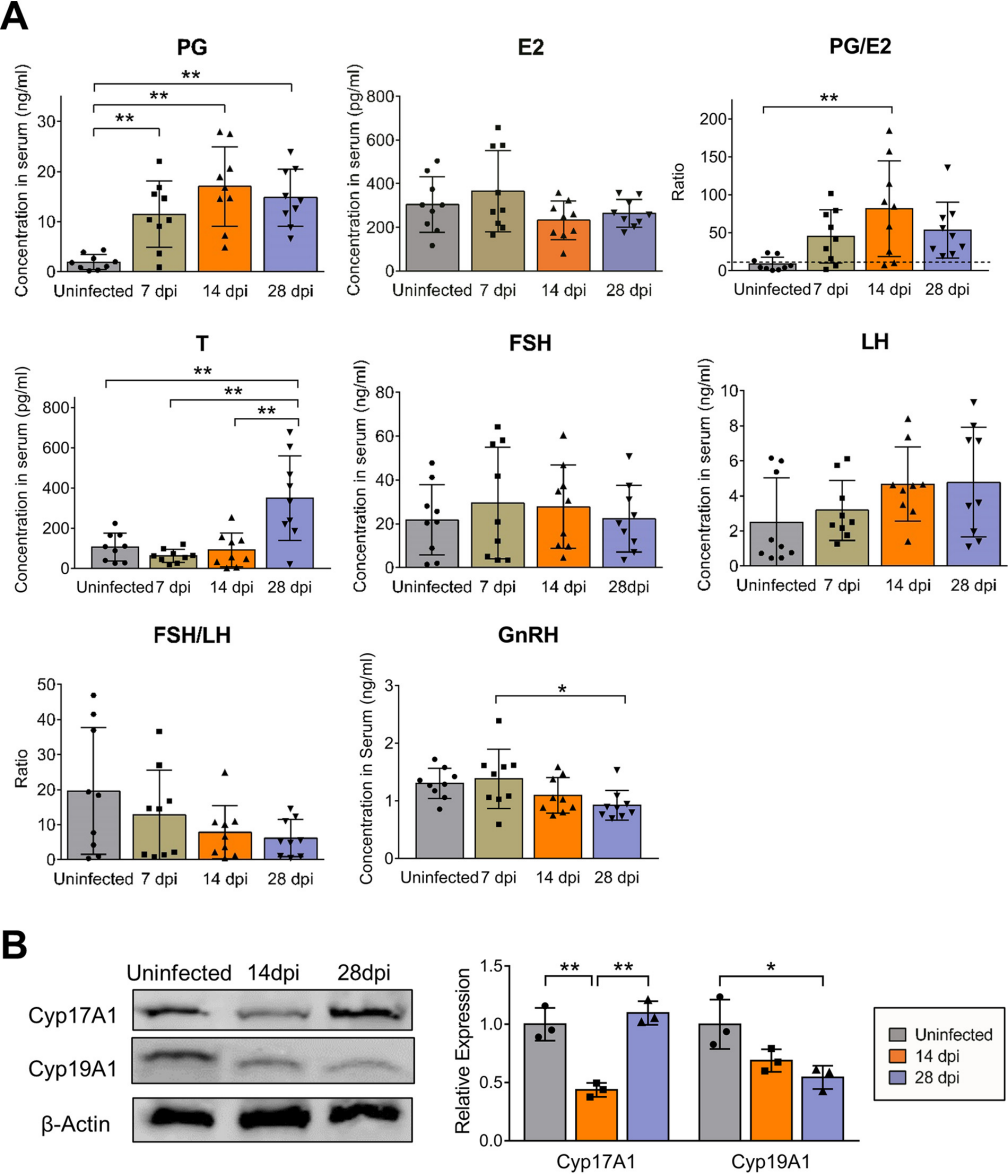
ZIKV infection causes disordered ovarian steroidogenesis. Adult female *Ifnar1^−/−^* mice 6 to 8 weeks old were subcutaneously inoculated with 10^4^ PFU ZIKV SMGC-1. Serum and ovaries were collected at different time points as indicated. (A) The levels of sex hormones and upstream regulatory hormones in the serum after ZIKV infection were determined by ELISA kits. Uninfected mice were considered controls. PG, progesterone; E2, estradiol; T, testosterone; FSH, follicle-stimulating hormone; LH, luteinizing hormone; GnRH, gonadotropin-releasing hormone (*n* = 9/group). (B) The expression levels of Cyp17A1 and Cyp19A1 in ZIKV-infected ovaries were determined at 14 and 28 dpi by Western blotting (left panels). Uninfected ovaries were considered controls. The relative expression levels of Cyp17A1 and Cyp19A1 (target proteins/β-actin) were further analyzed by measuring band density (right panel; *n* = 3/group). The data are presented as the means ± SD. Each spot represents an individual mouse. The significance of differences was analyzed by ordinary one-way ANOVA with a Tukey’s multiple-comparison test (A, B). *, *P* < 0.05; **, *P* < 0.01.

Because sex hormones are primarily synthesized in the ovaries of females, serum hormone levels, including the levels of progesterone, estradiol, and testosterone, were detected to evaluate ovarian function (9, 33). Surprisingly, compared with the control level (1.90 ± 1.50 ng/mL), the progesterone levels increased slightly at 7 dpi, increased significantly by approximately 9 times (17.05 ± 7.90 ng/mL) at 14 dpi, and remained at a high level (13.67 ± 6.54 ng/mL) at 28 dpi (Fig. 9A). The testosterone level did not change much in the early stage (62.47 ± 32.33 pg/mL at 7 dpi and 92.02 ± 79.21 pg/mL at 14 dpi) compared with that (106.26 ± 69.13 pg/mL) in uninfected mice, but it obviously increased to 349.7 ± 198.2 pg/mL at 28 dpi. However, there was no significant alteration in the estradiol level during the whole infection course, which made the ratio of progesterone to estradiol (PG/E2) in the serum increase to 45.11 ± 35.14 at 7 dpi compared with 8.69 ± 8.97 in uninfected mice, and the PG/E2 ratio was persistently maintained at a high level (81.61 ± 59.52 at 14 dpi and 53.37 ± 34.72 at 28 dpi) during infection (Fig. 9A). These changes could be associated with the pathological observations mentioned above (Fig. 4).

As upstream regulatory organs, the hypothalamus and pituitary control ovarian functions by secreting gonadotropin releasing hormone (GnRH) or follicle-stimulating hormone (FSH) and luteinizing hormone (LH), respectively (34, 35). We detected the serum levels of these hormones by enzyme-linked immunosorbent assay (ELISA). The results showed no obvious changes in the levels of FSH and LH during ZIKV infection. In contrast, the GnRH level displayed a reduced trend at 14 dpi (1.10 ± 0.31 ng/mL versus 1.30 ± 0.26 ng/mL in uninfected mice) and was decreased at 28 dpi (0.92 ± 0.26 ng/mL), which might result from negative feedback inhibition mediated by the increased downstream progesterone and testosterone levels (Fig. 9A).

Given that CYP17A1 and CYP19A1, mainly expressed in theca cells and in granulosa cells separately, are key enzymes for catalyzing the conversion of progesterone into testosterone and testosterone into estradiol, respectively (36, 37), the expression of CYP17A1 and CYP19A1 in the ovary was detected to investigate the mechanism underlying ZIKV-induced hormone disorder. The results showed that both CYP17A1 and CYP19A1 levels were obviously downregulated at 14 dpi. At 28 dpi, the CYP17A1 level had almost recovered to normal, while the CYP19A1 level remained below the normal level, which might be in line with the increased testosterone level observed at the same time (Fig. 9B).

**FIG 10:**
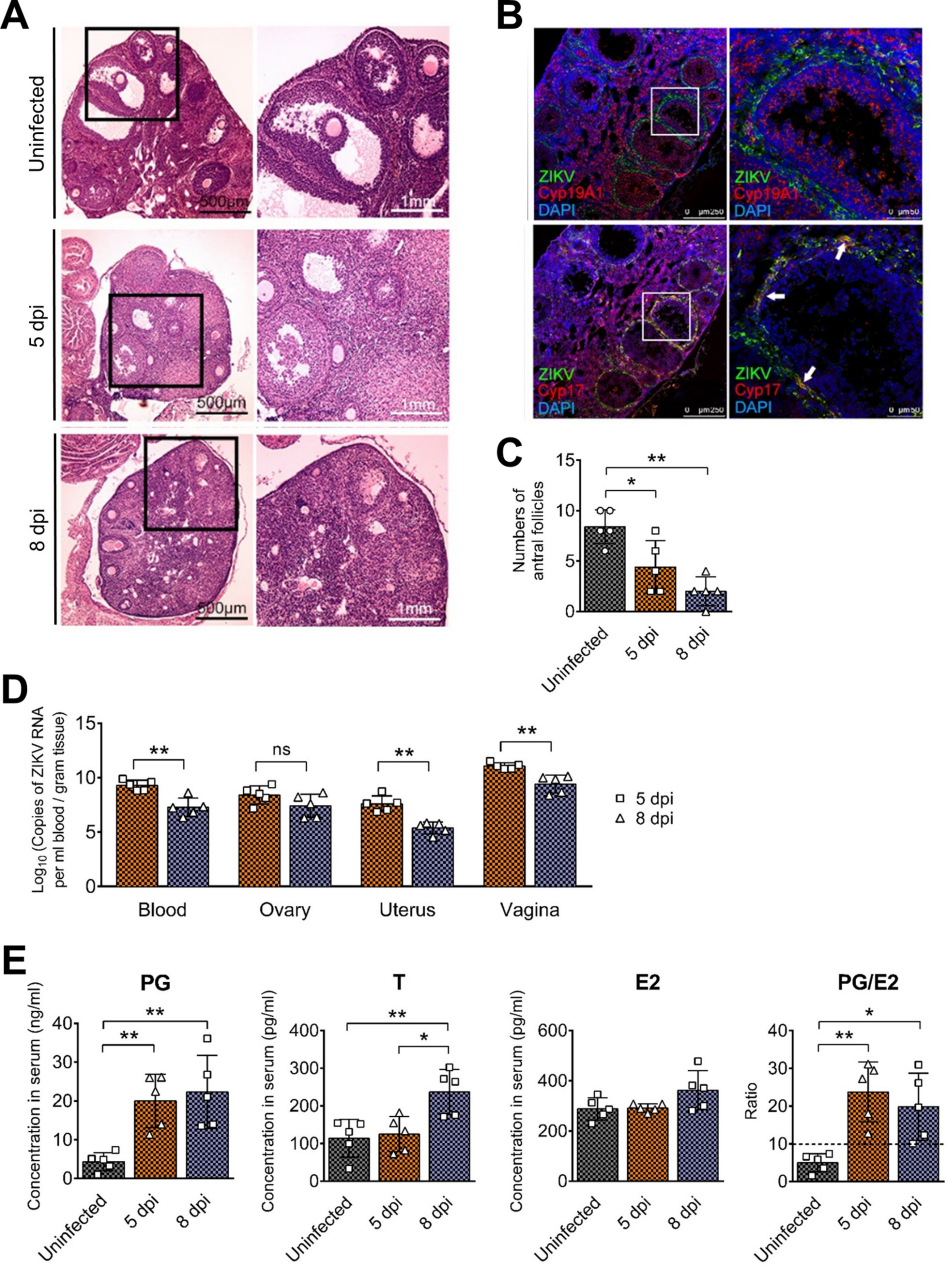
ZIKV disrupts the architecture and function of the ovaries in *Ifnar1^−/−^Ifngr1^−/−^* mice. Adult female *Ifnar1^−/−^Ifngr1^−/−^* mice 6 to 8 weeks old were subcutaneously inoculated with 10^4^ PFU ZIKV SMGC-1. Blood, serum, ovaries, uteri, and vaginas were collected at 5 and 8 dpi. (A) The histopathological changes in the ovaries of uninfected and ZIKV-infected *Ifnar1^−/−^Ifngr1^−/−^* mice at 5 and 8 dpi were analyzed by HE staining. (B) The distribution of ZIKV in the ovaries at 5 dpi was detected by IFA. ZIKV-positive cells are shown in green, Cyp17A1 staining for theca cells or Cyp19A1 staining for granulosa cells is shown in red, and DAPI staining for nuclei is shown in blue. Cyp17A1, cytochrome P450 17A1; Cyp19A1, aromatase. (C) Numbers of antral follicles were calculated (*n* = 5/group). (D) The viral RNA loads in the blood, ovaries, uteri, and vaginas at 5 and 8 dpi were analyzed by qRT-PCR (*n* = 5/group). (E) The levels of sex hormones in the serum after ZIKV infection were determined by ELISA kits. Uninfected mice were considered controls. PG, progesterone; E2, estradiol; T, testosterone (*n* = 5/group). The data are presented as the means ± SD. Each spot represents an individual mouse. The significance of differences was evaluated by ordinary one-way ANOVA with a Tukey’s multiple-comparison test (C, E) and the Mann-Whitney test (D). *, *P* < 0.05; **, *P* < 0.01. ns, no significant difference.

We also infected *Ifnar1^−/−^Ifngr1^−/−^* mice with ZIKV to verify this pathogenic characteristic of ZIKV. As expected, the results were similar to those found in *Ifnar1^−/−^* mice, including the loss of antral follicles and markedly augmented progesterone and testosterone levels (Fig. 10). These results further confirmed the specific effects of ZIKV on ovarian function.

### ZIKV infection disrupts the estrous cycle and compromises conception.

**FIG 11:**
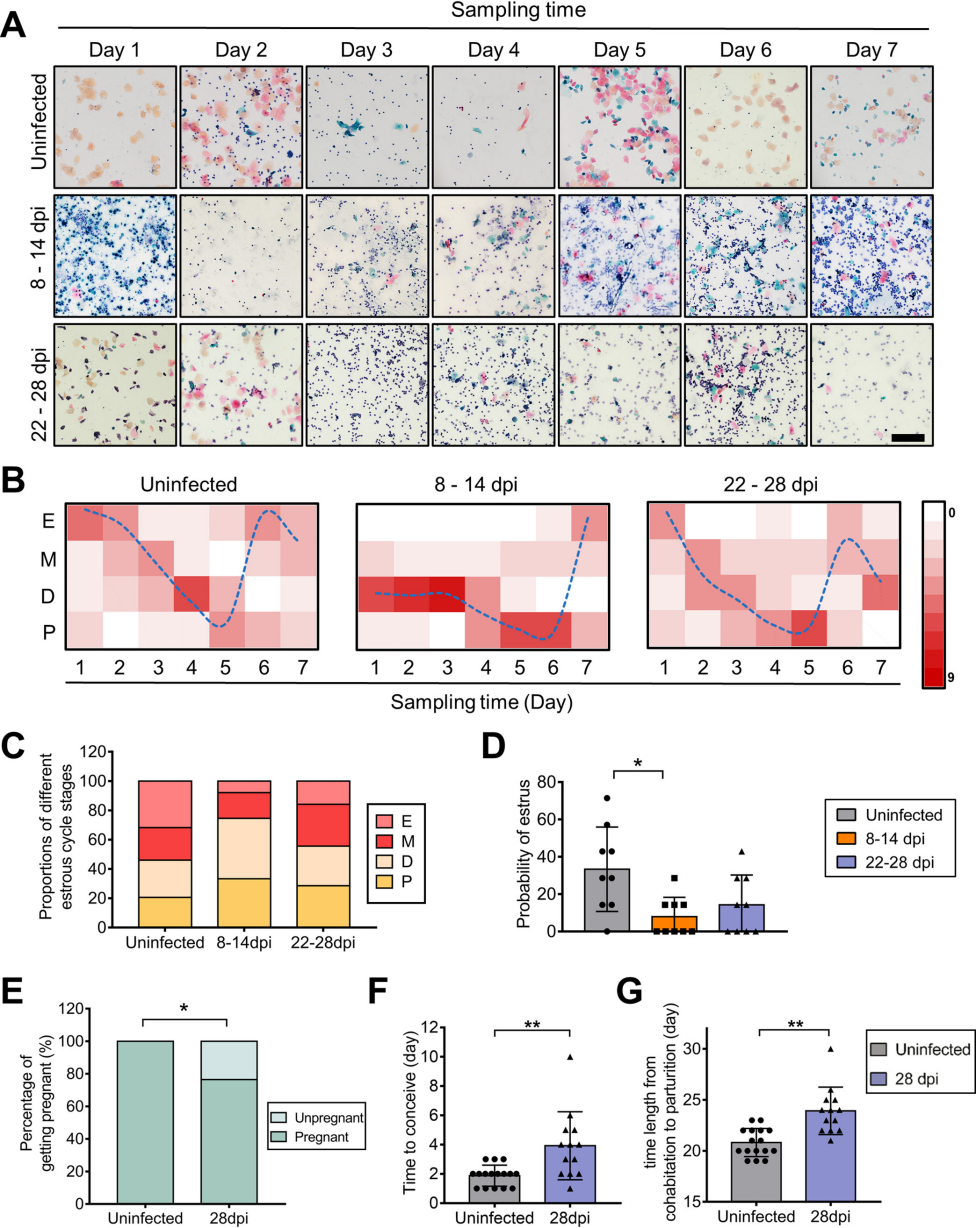
ZIKV infection disturbs the estrous cycle. Adult female *Ifnar1^−/−^* mice 6 to 8 weeks old were subcutaneously inoculated with 10^4^ PFU ZIKV SMGC-1. Serial vaginal smears were sampled daily during indicated periods after ZIKV infection. The stages of the estrous cycle were determined by Papanicolaou staining. (A) Representative images of dynamic changes in the estrous cycle in an uninfected and ZIKV-infected *Ifnar1^−/−^* mice at 8–14 dpi and 22–28 dpi. Bar = 200 μm. (B) Fitting curves of periodic changes in the estrous cycle in uninfected and ZIKV-infected *Ifnar1^−/−^* mice at 8 to 14 days and 22 to 28 days (*n* = 9/group). E, estrus; M, metestrus; D, diestrus; P, proestrus. (C) The proportions of different stages of the estrous cycle in examined samples (*n* = 9/group). (D) The probability of estrus in *Ifnar1^−/−^* mice with or without ZIKV infection (*n* = 9/group). (E to G) Impacts of ZIKV on the capacity to conceive in mice. ZIKV-infected mice at 28 dpi and uninfected mice were separately cohabited with uninfected male *Ifnar1^−/−^* mice for 10 days. The pregnancy rate (E), the time to conceive after cohabiting (F), and the time length from cohabitation to parturition (G) were recorded (*n* = 16 to 17/group). The data are presented as the means ± SD. Each spot represents an individual mouse. The significance of differences was evaluated by Kruskal-Wallis test with a Dunn’s multiple-comparison test (D), the chi-square test (E), and the Mann-Whitney test (F and G). *, *P* < 0.05; **, *P* < 0.01.

Normally, ovarian sex hormones directly regulate the estrous cycle (estrus, metestrus, diestrus, and proestrus) in mice, which is strongly associated with the potential for conception. To address this question, vaginal smears were collected from virgin female *Ifnar1^−/−^* mice daily from 8 to 14 dpi and from 22 to 28 dpi with ZIKV. The stage of the estrous cycle was monitored by vaginal cytology according to general criteria (15, 38). In uninfected female mice, an estrous cycle lasting for approximately 5 days was periodically observed. However, after ZIKV infection, the duration was significantly prolonged, and the fluctuation of the estrous cycle was disturbed. Vaginal epithelial cornification almost disappeared between 8 and 14 dpi, demonstrating the decreased proportion of the estrus (Fig. 11A and B). From 22 to 28 dpi, the above changes gradually subsided, but the proportion of the estrus in the infected group was still lower than that in the uninfected groups, and the fluctuation of the estrous cycle and the probability of the estrus had not returned to normal (Fig. 11B to D). These results were consistent with the disordered sex hormone levels, especially the continuously elevated progesterone level.

To further evaluate the influences of ZIKV infection on conception capacity, uninfected or ZIKV-infected female virgin mice (28 dpi) were cohabited with healthy male *Ifnar1^−/−^* mice for 10 days. Processes were monitored daily. All the uninfected female mice became pregnant in 3 days, with an average time to conceive of 1.88 days. However, in the ZIKV-infected mice, the pregnancy rate dropped to 76.47% even the time length of cohabitation was extended to 10 days, and the average time to conceive was prolonged to 3.92 ± 2.23 days (Fig. 11E and F), significantly longer than the uninfected mice. Among them, time to conceive more than 3 days was observed in about 50% of mice. Additionally, the time length from cohabitation to parturition was also significantly prolonged (Fig. 11G). These indicated the inhibited estrus and diminished conception capacity after ZIKV infection, which likely resulted from the disordered sex hormone levels reflected by cornification changes in the vaginal epithelium.

### ZIKV has long-term adverse impacts on the ovary, even after viral clearance.

**FIG 12:**
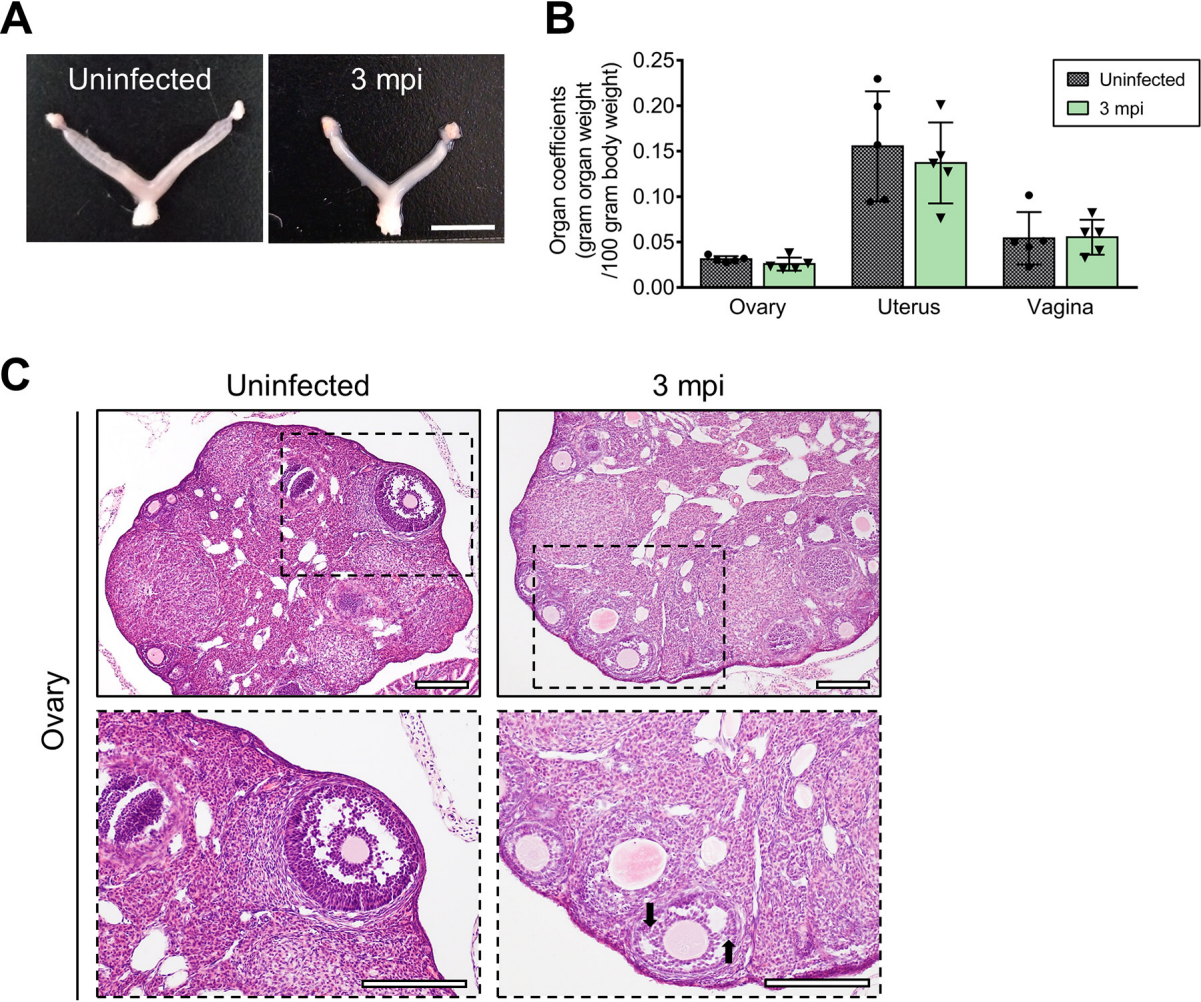
Histopathological changes in the ovary at 3 mpi. Adult female *Ifnar1^−/−^* mice 6 to 8 weeks old were subcutaneously inoculated with 10^4^ PFU ZIKV SMGC-1. Ovaries, uteri, and vaginas were collected at 3 months postinfection (mpi). Uninfected age-matched mice were considered controls. (A) Representative morphological changes in the female genital organs of uninfected and ZIKV-infected *Ifnar1^−/−^* mice at 3 mpi. Bar = 5 mm. (B) The organ coefficients (g organ weight/100 g body weight) of the ovaries, uteri, and vaginas of uninfected mice and ZIKV-infected mice at 3 mpi (*n* = 5/group). (C) Histopathological changes in the ovaries of uninfected mice and ZIKV-infected mice at 3 mpi. Bar = 200 μm. Loosely arranged granulosa cell layers were indicated by arrows.

**FIG 13:**
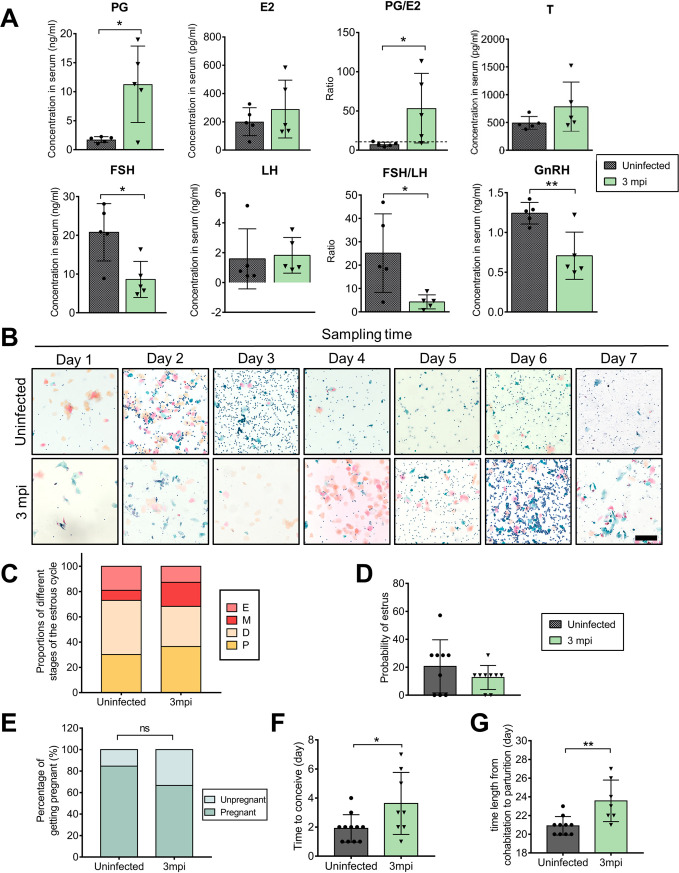
Long-term impacts of ZIKV infection on reproductive endocrine disorder. Adult female *Ifnar1^−/−^* mice 6 to 8 weeks old were subcutaneously inoculated with 10^4^ PFU ZIKV SMGC-1. Serum was collected at 3 months postinfection (mpi). Uninfected age-matched mice were considered controls. (A) Levels of sex hormones and upstream regulatory hormones in the serum at 3 mpi. Serum samples from uninfected mice served as controls. PG, progesterone; E2, estradiol; T, testosterone; FSH, follicle-stimulating hormone; LH, luteinizing hormone; GnRH, gonadotropin-releasing hormone (*n* = 5/group). (B) Representative images of dynamic changes in the estrous cycle in an uninfected and a ZIKV-infected *Ifnar1^−/−^* mouse at 3 mpi. Vaginal smears were collected daily and stained by the Papanicolaou method. Bar = 200 μm. (C) The proportions of different stages of the estrous cycle in examined vaginal smears. E, estrus; M, metestrus; D, diestrus; P, proestrus (*n* = 9/group). (D) The probability of estrus in *Ifnar1^−/−^* mice with or without ZIKV infection (*n* = 9/group). (E to G) Impacts of ZIKV on the capacity to conceive in mice. ZIKV-infected mice at 3 mpi and uninfected mice were separately cohabited with uninfected male *Ifnar1^−/−^* mice for 10 days. The pregnancy rate (E), the time to conceive after cohabiting (F), and the time length from cohabitation to parturition (G) were recorded (*n* = 12 to 13/group). The data are presented as the means ± SD. Each spot represents an individual mouse. The significance of differences was evaluated by Mann-Whitney test (D, F, and G) and the chi-square test (E). *, *P* < 0.05; **, *P* < 0.01.

Next, the long-term influences of ZIKV infection on ovarian functions were investigated. At 3 months postinfection (mpi), ZIKV RNA was undetectable in the ovaries, uterus, or vagina of all five observed mice in 40 cycles by real-time quantitative PCR (RT-qPCR), whereas the average cycle threshold value for positive control was 16.54. Meanwhile, we did not observe any positive staining of viral antigens by IFA. These results suggested that ZIKV was eliminated completely. Consistently, the organ coefficients of these three tissues in infected mice were almost recovered to corresponding levels in uninfected mice (Fig. 12A and B). However, compared with acute ZIKV infection, the phase after ZIKV clearance showed that the granulosa cell layers of antral follicles were still thinner and the arrangement of the cells was looser, although the ovarian histopathological changes had partially recovered, and the numbers of antral follicles were developed, indicating potential defects in follicles (Fig. 12C). Interestingly, the levels of progesterone and testosterone remained significantly augmented, and the estradiol level changed little. For upstream regulatory hormones, the LH level did not change obviously, but the FSH and GnRH levels were markedly decreased (Fig. 13A). These results indicated that abnormalities in reproductive hormones persisted even after viral clearance.

Noticeably, the proportion of the estrus was still lower at 3 mpi, as revealed by assessing exfoliated vaginal cells (Fig. 13B to D). Then, uninfected or ZIKV-infected virgin mice were cohabited with healthy male *Ifnar1^−/−^* mice for 10 days to observe the long-term effects of ZIKV on conception capacity. The results showed that 84.61% (11 of 13) of the uninfected female mice became pregnant in 4 days with an average time to conceive of 1.91 days. In contrast, the pregnancy rate was only 66.67% (8 of 12), with an extended average time to conceive of 3.63 ± 2.00 days and significantly prolonged time length from cohabitation to parturition in the ZIKV-infected mice (Fig. 13E to G). Taken together, our results suggested that the morphology and functions of the ovary were not fully recovered from ZIKV infection even after viral clearance.

### DENV-2 do not infect the ovaries.

**FIG 14:**
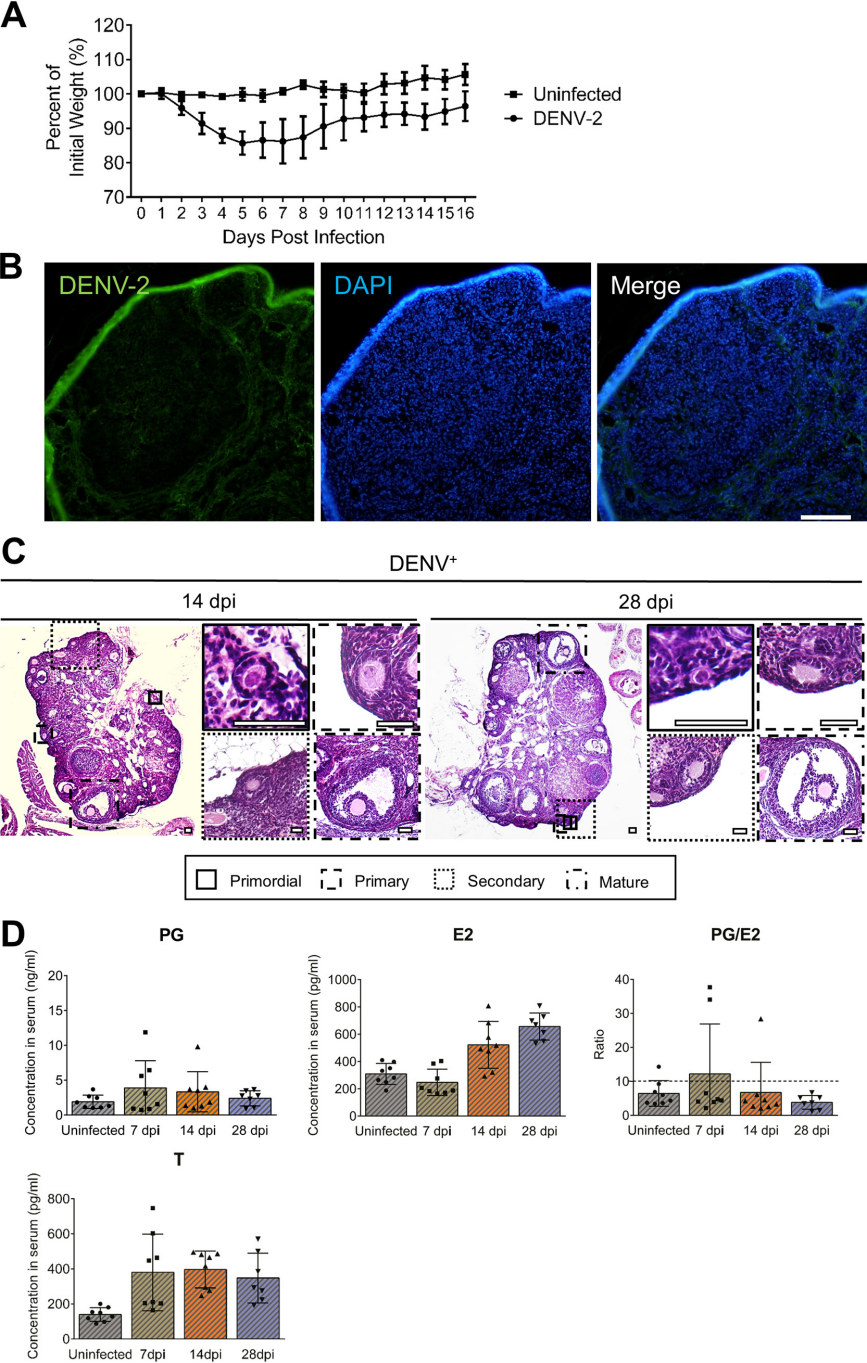
DENV-2 does not infect the ovary and alter the ovarian architecture or function. Adult female *Ifnar1^−/−^* mice 6 to 8 weeks old were subcutaneously inoculated with 10^5^ PFU DENV-2 Tr1751. The serum and ovaries were collected at indicated time points. Uninfected mice were considered controls. (A) The body weights of DENV-infected and uninfected mice were monitored over time (*n* = 8/group). (B) The distribution of DENV-2 in the ovaries at 28 dpi was detected by IFA. DENV-positive cells were shown in green, and DAPI staining for nuclei was shown in blue. Bar = 50 μm. (C) The histopathological changes in the ovaries of DENV-infected mice were analyzed at 14 and 28 dpi by HE staining. Bar = 50 μm. (D) The levels of sex hormones in the serum after DENV-2 infection were determined by ELISA kits. PG, progesterone; E2, estradiol; T, testosterone (*n* = 7 to 8/group). The data are presented as the means ± SD. Each spot represents an individual mouse.

To investigate whether the effects of ZIKV infection on the ovary are specific, adult female *Ifnar1^−/−^* mice were subcutaneously inoculated with 10^5^ PFU DENV-2 (strain Tr1751). The results showed that the infected mice gradually lost body weight after infection, and the lowest body weights were observed between 5 and 7 dpi (Fig. 14A). However, after DENV-2 infection, no viral antigens were detected in the ovaries (Fig. 14B), further suggesting that the ovary was a target organ for ZIKV. Unlike in ZIKV-infected mice, the histomorphology of the ovaries in DENV-2-infected mice displayed little change (Fig. 14C).

To further validate that the endocrine change was caused by ovarian infection rather than general infection, the levels of progesterone, estradiol, and testosterone in the serum were measured to determine the changes in sex hormones after DENV-2 infection. Although the estradiol and testosterone levels were slightly elevated, the progesterone level remained unchanged, and the ratio of PG/E2 showed no change during the whole infection course (Fig. 14D). These results illustrated that ZIKV infection but not DENV-2 infection significantly induced disordered ovarian steroidogenesis, especially markedly augmented progesterone expression.

**FIG 15:**
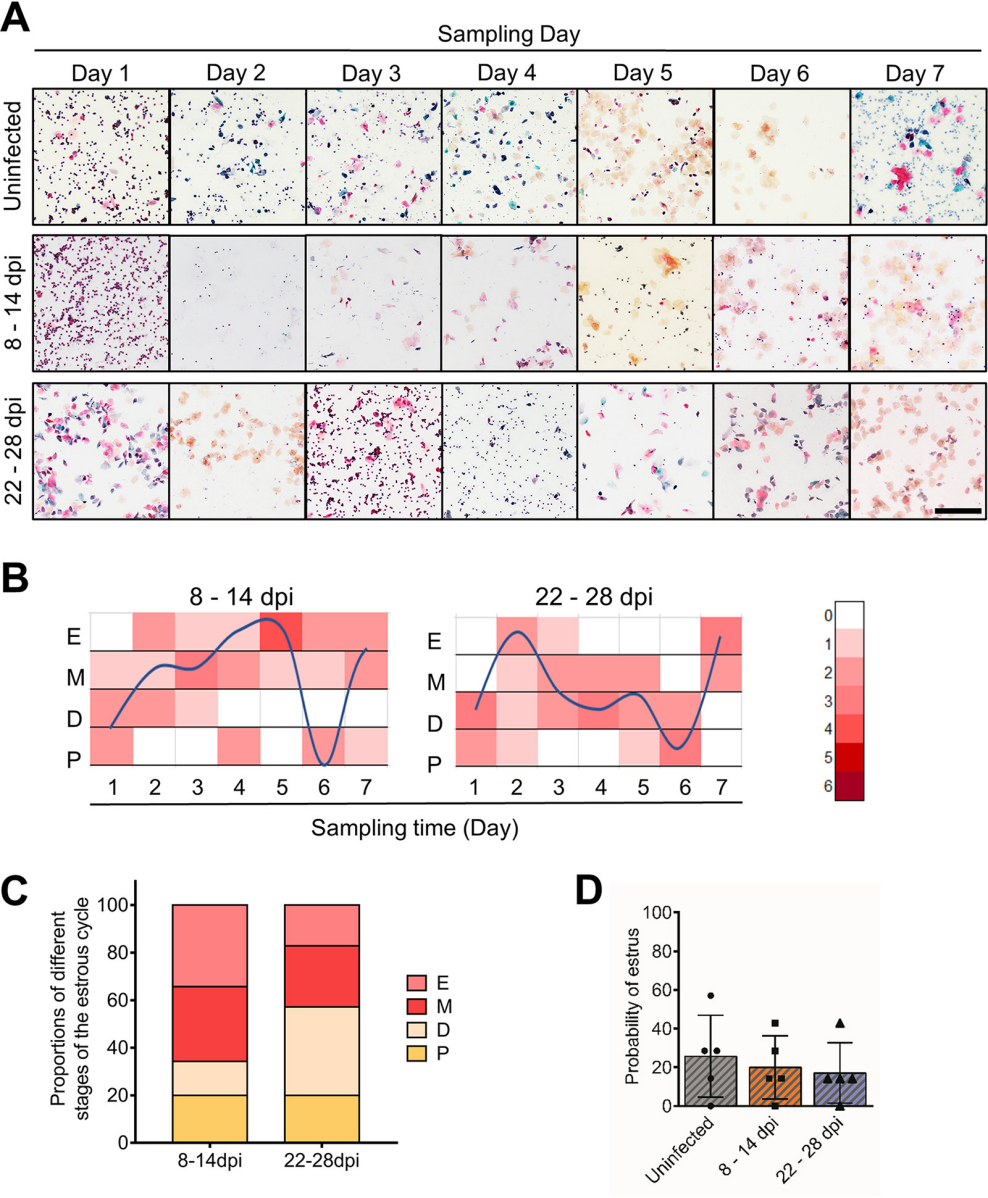
DENV-2 infection has little influence on the estrous cycle. Adult female *Ifnar1^−/−^* mice 6 to 8 weeks old were subcutaneously inoculated with 10^5^ PFU DENV-2 Tr1751. Serial vaginal smears were sampled daily during indicated periods after DENV-2 infection. The stages of the estrous cycle were determined by Papanicolaou staining. (A) Representative images of the dynamic changes in the estrous cycle in an uninfected *Ifnar1^−/−^* mouse and DENV-infected mice at 8 to 14 dpi and 22 to 28 dpi. Bar = 200 μm. (B) Fitting curves of periodic changes in the estrous cycle of DENV-infected mice at 8 to 14 dpi and 22 to 28 dpi. (C) The proportions of different stages of the estrous cycle in examined samples. E, estrus; M, metestrus; D, diestrus; P, proestrus (*n* = 5/group). (D) The probability of estrus in *Ifnar1^−/−^* mice with or without DENV-2 infection (*n* = 5/group). The data are presented as the means ± SD. Each spot represents an individual mouse.

The stage of the estrous cycle was also monitored in DENV-2-infected mice. In comparison, after DENV-2 infection, the fluctuation of the estrous cycle remained regular, and the proportion of the estrus remained normal (Fig. 15). These results indicated the limited influences of DENV-2 infection on the ovary, further confirming that the pathogenicity of ZIKV in the ovary is specific.

## DISCUSSION

To date, ZIKV has been indicated to be the only flavivirus that can be transmitted by sexual and vertical routes. Numerous studies have demonstrated that ZIKV persistently replicates in the male genital tract and damages male fertility in mouse models and human testicular cell culture systems. However, there have been few studies on ZIKV infection in the female gonads (39). More importantly, the effects of ZIKV infection on female reproductive health remain unclarified. Because the ovary is a multicompartmental and dynamic organ that plays a key role in female reproductive health, the influences of ZIKV on the ovary urgently need to be elucidated.

In this study, we demonstrate that ZIKV can replicate in the ovary, with infectious ZIKV particles isolated in the acute phase of infection and persistently high RNA levels detected. The disrupted ovarian architecture in the ZIKV-infected nonpregnant *Ifnar1^−/−^* mice indicates that the ovary is an important target for ZIKV, but not for DENV-2. Morphological changes in granulosa cells and oocytes of antral follicles and significantly decreased number of antral follicles in ZIKV-infected ovaries suggest possible ovarian retrogression. Because folliculogenesis requires carefully orchestrated cross talk between an oocyte and the surrounding granulosa cells, the elevated apoptotic granulosa cells may accelerate follicular atresia, which indicates that ZIKV infection accelerates antral follicle loss.

GDF-9, EPAB, and BMP-15 expressed by oocytes or granulosa cells are major positive regulatory factors in folliculogenesis from primary to antral follicles, while AMH is produced by the granulosa cells of growing follicles and acts as an inhibitor in this process (40). Expression of these genes is required for the development of follicles through different stages. In this study, we found that the significantly decreased mRNA levels of GDF-9, EPAB, and BMP-15 and unaltered AMH after ZIKV infection can impede follicular development to the antral stage. The decreased antral follicle numbers after ZIKV infection could be closely associated with these two processes.

The synthesis of sex hormones is another important ovarian function, and stable sex hormone levels are crucial to maintaining not only reproductive health but also normal physiological function in females. The steroid hormones are synthesized in the growing follicles and the terminally differentiated luteal cell in the ovary (11, 12). Given that ZIKV replicates in theca cells and granulosa cells and ZIKV infection impedes follicular development, we detected the serum levels of sex hormones to reflect the effects of ZIKV on ovarian steroidogenesis. Continuously increased progesterone level and subsequently increased testosterone level indicates that ZIKV infection induces disordered sex hormone levels. In combination with the limited changes in upstream regulatory hormones, our results suggest that ZIKV specifically affects ovarian function.

To uncover the underlying mechanisms involved in sex hormone disorder, we next assessed the expression of key enzymes linked to ovarian steroidogenesis. Downregulated levels of CYP17A1 and CYP19A1 in ZIKV-infected ovaries may be the key reason for the continuously increased progesterone and testosterone levels. However, the two downregulated enzymes did not recover simultaneously. This difference may lead to the lingering peak observed for the testosterone level compared to the progesterone level after ZIKV infection. In addition, we observed significantly decreased numbers of the corpus luteum accompanied by an abnormal increase in the volume of the corpus luteum. Whether this is another important reason why the progesterone levels keep rising requires more research. Additionally, in this study, the levels of FSH, LH, and GnRH, which can regulate downstream sex hormones, did not change obviously during the early stage after infection. These results suggest that ZIKV infection in the ovary disrupts normal ovarian function, as revealed by hormonal disorder.

The estrous cycle is tightly regulated by sex hormones and can directly reflect changes in sex hormones in mice. In this study, we found that ZIKV infection but not DENV-2 infection obviously decreased the proportion of the estrous and caused an irregular estrous cycle, indicating that it was difficult for ZIKV-infected mice to become pregnant. Additionally, the prolonged time to conceive and lower pregnancy rate suggested that the capacity to conceive was negatively impacted by ZIKV infection. These results were in line with the persistently high progesterone level in the serum. However, it is worth mentioning that, regardless of irregular periodic changes in different normal individuals, the tendencies of pathological changes, including those determined by assessing histopathology, viral loads, and hormone levels, appeared similar after ZIKV infection in our study. The results indicate that the tropism of ZIKV for the ovary was almost equivalent in all stages.

To understand the long-term effects of ZIKV on the ovary, hormone levels, the estrous cycle, and the capacity to conceive were further investigated at 3 months after ZIKV infection. Surprisingly, progesterone and testosterone levels were sustained at high levels and accompanied by markedly decreased levels of the upstream hormones FSH and GnRH, which might be attributed to negative feedback inhibition of downstream progesterone and testosterone or ZIKV replication in the hypothalamus (41). Consistently, the proportion of the estrus and the capacity to conceive were still lower in the mice in the infected group than in those in the uninfected group. These results indicate that the influences of ZIKV infection on the ovary can persist for a long time even after virus elimination.

A recent study reported by Caine et al. assessed the impact of ZIKV on female fertility by testing the oocytes of mice for morphology and *in vitro* fertilization (IVF) capacity and found no long-term (90 dpi) consequences of ZIKV infection on ovarian follicular reserve or fertility (5). These results are consistent with our results *in vivo* about no significant difference in pregnancy rate at 3 mpi. In fact, pregnancy success refers to the whole process from mating to the birth of the pups. Our study was more concerned about the effects of ZIKV on the process before conception and the conception processes in nonpregnant mice, which was closely related to ovarian endocrine function. However, our results were obtained by using immunocompromised mice, and ovarian function and histopathology might be affected to a greater extent than in humans during ZIKV infection. Thus, these results cannot simply be used to analyze human diseases. The continuously elevated progesterone level and compromised conception caused by ZIKV infection in mice do provide clues for the related follow-up studies.

As shown for the testis, an important proven target of ZIKV, the effects of ZIKV on male reproductive functions are much worse than those on female reproductive functions, as revealed by morphological changes, hormone disorders, and local inflammation (42–44). This may be attributed to periodic and spontaneous ovarian ovulation and follicular atresia, which may contribute to viral clearance. In males, sperm are produced in the testes, stored in the epididymides, and expelled only during mating, possibly creating a greater opportunity for ZIKV maintenance *in situ*. Further studies are required to clarify this issue. Moreover, unlike orchitis, oophoritis is a poorly diagnosed complication of virus infection, attributed to the anatomical position of the ovaries. Oophoritis may mimic the acute abdominal pain of appendicitis, rendering clinical diagnosis difficult (45). Therefore, the sex hormone disorder and abnormal ovarian functions caused by ZIKV infection in mice may have important implications for women of reproductive age.

**FIG 16:**
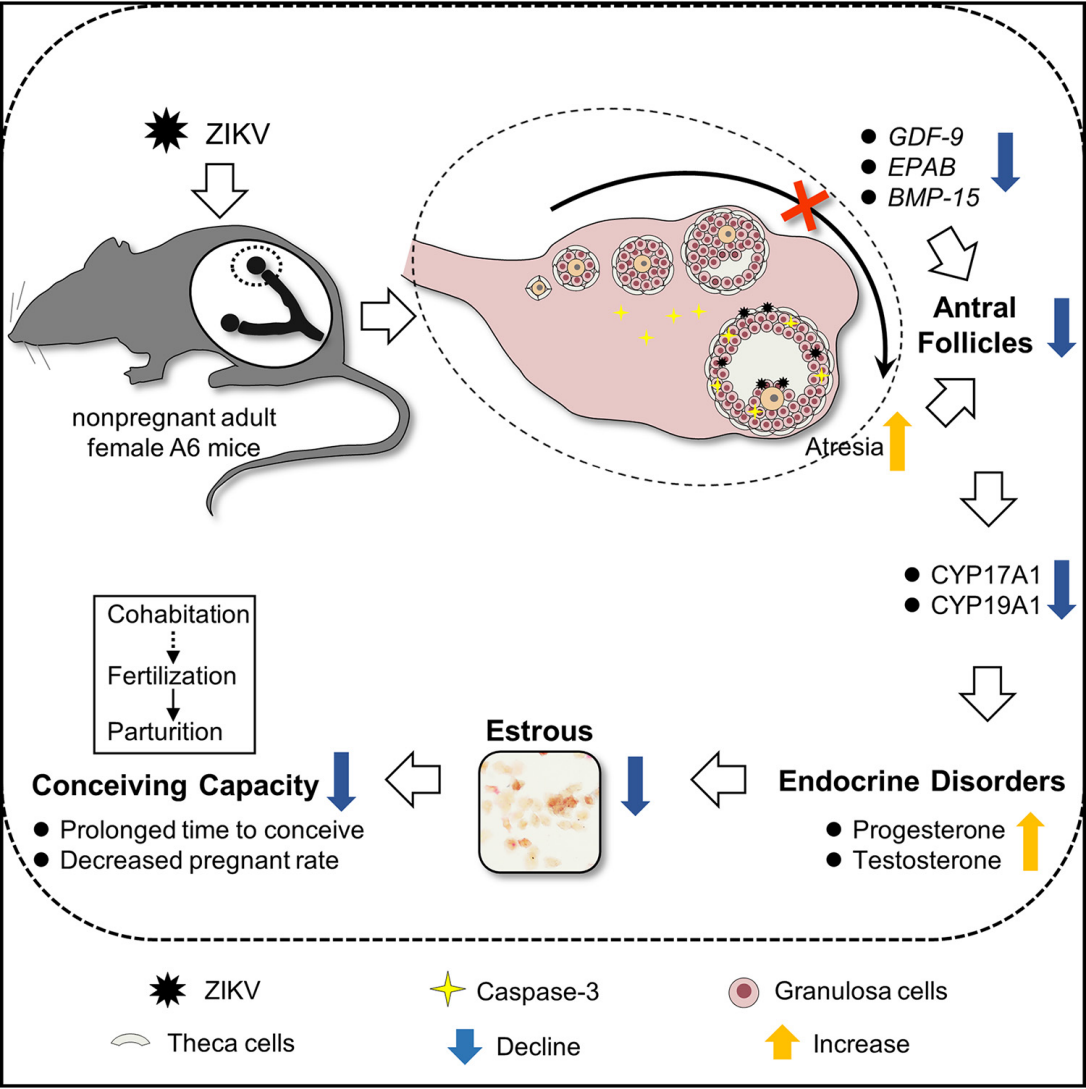
Schematic diagram to show the pathogenesis of ZIKV infection in the ovary. ZIKV infection in nonpregnant female mice leads to ovarian damage and dysfunction manifested by the decreased numbers of antral follicles and disordered ovarian steroidogenesis. The endocrine dyscrasia disrupts the estrous cycle and prolongs the time to conceive in mice.

In conclusion, our study demonstrates that the ovary efficiently supports ZIKV replication, and the theca cells and granulosa cells were susceptible to ZIKV in mice. ZIKV infection in ovarian granulosa cells leads to apoptosis, which accelerates follicular atresia. Moreover, the folliculogenesis of ZIKV-infected ovaries is significantly impeded, which is likely linked to the decreased numbers of antral follicles and indicates ovarian degeneration. Furthermore, ZIKV infection in theca cells and granulosa cells significantly disrupts enzymes related to steroidogenesis, which leads to endocrine disorders, especially a continuously increased serum level of progesterone. This disorder markedly reduces the occurrence of estrus and interferes with the conception capacity of mice. More importantly, even after ZIKV clearance, endocrine dyscrasia and reduced estrus do not completely recover (Fig. 16). This study indicates adverse effects of ZIKV infection on the ovaries of mice, but further prospective studies in ZIKV-infected nonhuman primates and women are needed to corroborate these findings.

## MATERIALS AND METHODS

### Ethics statement.

All the animal experimental protocols were approved by and conducted in accordance with the guidelines established by the Institutional Animal Care and the Animal Ethics Committees of Capital Medical University, Beijing, China.

### Cells, viruses, and mice.

The Aedes albopictus mosquito cell line (C6/36) used for ZIKV (strain SMGC-1, Asian lineage, GenBank accession number KX266255), which was isolated from an imported case returning to China from Fiji and Samoa at the Shenzhen Port (46) and provided by George Fu Gao (47), or DENV-2 (strain Tr1751) propagation was cultured in RPMI 1640 medium supplemented with 10% fetal bovine serum (FBS; Gibco). Vero cells, which were used to determine the titer of ZIKV by a plaque assay with overlay medium containing 1.2% methylcellulose, were maintained in minimum essential medium (MEM) supplemented with 5% FBS. None of the cell lines had mycoplasma contamination. ZIKV stocks were stored at −80°C until use.

Pregnant BALB/c mice were purchased from the Academy of Military Medical Sciences (Beijing, China). C57BL/6 mice deficient in interferon α/β receptors (*Ifnar1^−/−^* mice) were purchased from the Institute of Zoology, Chinese Academy of Sciences. C57BL/6 mice deficient in interferon α/β and γ receptor (*Ifnar1^−/−^Ifngr1^−/−^* mice) were bred in our laboratory. The mice were maintained on an automatic light cycle (12 h light:12 h dark) in a specific pathogen-free animal facility. For ZIKV infection, female *Ifnar1^−/−^* or *Ifnar1^−/−^Ifngr1^−/−^* mice 6 to 8 weeks old were used and challenged with 10^4^ PFU ZIKV through footpad injection, and an equal volume of phosphate-buffered saline (PBS) was used as a control. Survival indexes of mice, including body weights and survival rates, were recorded daily for up to 28 days. Once the mice had been euthanized, we recorded the body weights and then weighed the ovary, uterus, and vagina by an analytical balance. The organ coefficient was generally determined by the weight of an organ (wet weight) per 100 g of body weight (48). Additionally, samples, including the serum, reproductive tissues, were collected at 14 or 28 dpi or 3 mpi for determination of hormone levels, viral loads, and cytokine levels and histological examination.

In pregnancy experiments, females were cohabited with males of proven fertility in the afternoon, and female mice were examined for vaginal plugs next morning. If the vaginal plug was observed, that day was considered first day of pregnancy. Pregnancy success was defined as pregnancies brought to term (successful delivery of live litters) in all female mice with vaginal plugs.

### RT-qPCR.

Snap-frozen tissues extracted from female *Ifnar1^−/−^* mice were pulverized with a series of sharp blows in lysing tubes containing TRIzol (Sigma) in a homogenizer instrument (Next Advance). Total RNA was isolated from the homogenized TRIzol lysates according to the manufacturer’s protocol. The levels of ZIKV RNA and mRNA transcripts for folliculogenesis-related genes were determined by one-step quantitative RT-PCR using the Quant one-step RT-qPCR kit (SYBR green, Tiangen, China) and 7500 real-time PCR system (Applied Biosystems). The primer sets for folliculogenesis-related genes were kept the same with the published method (49, 50). The specificity of these primer sets has been validated by alignment at the NCBI, and the PCR amplification products were electrophoresed and sequenced. Viral genomic copies were normalized by a standard curve used to determine the limit of detection of the assay as previously reported (43). Log dilutions of a viral RNA standard template, kindly provided by Ai-hua Zheng at the Chinese Academy of Sciences (CAS), were similarly reverse transcribed, and 10-fold serial dilutions were used during each RT-qPCR amplification. ZIKV mRNA results are expressed as the copy number per g organ or per mL blood.

### ELISA and Luminex assays.

Mouse blood was harvested by tail bleeding for frequently repeated studies or by lancing the facial vein at the endpoint and centrifuged at 1,500 × *g* for 20 min. The serum was collected from the supernatant and stored at −80°C until analysis. ELISA kits were used to quantify the concentrations of PG, E2, T, LH, FSH, and GnRH (CEA459Ge, CEA461Ge, CEA458Ge, CEA441Mu, CEA830Mu, CEA843Mu, and Cloud-Clone) in mouse serum according to the manufacturer’s instructions based on the competitive ELISA technique. These kits were all qualified, and no significant cross-reactivity or interference between them and analogues were observed. The detection limit of progesterone (PG), estradiol (E2), testosterone (T), LH, FSH, and GnRH were 0.47 ng/mL, 4.45 pg/mL, 17.7 pg/mL, 145.2 pg/mL, 0.84 ng/mL, and 4.64 pg/mL, respectively. The interassay and intraassay variation coefficients for these kits were less than 12% and 10%, respectively. Briefly, 50 μl standards, blanks, and prediluted samples were added to precoated wells, followed immediately by the addition of 50 μl detection reagent A. After incubation and washing, 100 μl prepared detection reagent B was added and incubated for 30 min at 37°C, and then 90 μl substrate solution was added for color development. Finally, 50 μl stop solution was added, and the light absorbance was read at 450 nm on a Multiskan spectrum 1500 (Thermo Scientific). The intensity of the developed color was inversely proportional to the concentration of the hormone in the serum, which was calculated from the standard curve. The levels of cytokines and chemokines in ovarian lysates were detected by Bio-Plex multiplex immunoassays according to the manufacturer’s instructions (Bio-Rad).

### Immunofluorescence (IF) staining.

Ovaries collected from ZIKV-infected or control mice at different time points were embedded immediately in optimal cutting temperature compound (OCT, SAKURA) to prepare frozen sections (6 μm in thickness) with a freezing microtome (Leica, Germany). To analyze ZIKV antigens and their potential locations, immunofluorescence double staining was conducted. The sections were incubated with a rabbit anti-cytochrome P450 17A1 antibody (CYP17A1, 1:300, Abcam, ab125022), a rabbit anti-aromatase antibody (CYP19A1, 1:300, Abcam, ab18995), or mouse anti-ZIKV polyclonal serum (1:200) as the primary antibody at 4°C overnight. The slides were then incubated for 1 h at room temperature with secondary antibodies, including Alexa Fluor 594 donkey anti-rabbit IgG (H+L) (1:1,000; catalogue no. A21207, Life Technologies) and Alexa Fluor 488 goat anti-mouse IgG (H+L) (1:1,000; catalogue no. A11001, Life Technologies). A coverslip was mounted on each slide with mounting medium containing DAPI (ZLI-9557, ZSGB-BIO, China), and the slides were then examined under a confocal laser microscope.

### Immunohistochemistry (IHC) staining.

To assess the activation of apoptosis in the ovaries after ZIKV infection, the sections were incubated with a rabbit polyclonal anti-cleaved caspase-3 antibody (1:300, Cell Signaling Technology, 9664S) or an isotype control antibody (1:300, Cell Signaling Technology, 3900S) at 4°C overnight. The slides were then stained with a secondary horseradish peroxidase (HRP)-conjugated goat anti-rabbit antibody (PV-9001, ZSGB-BIO) for 20 min at room temperature. The 3,3′-diaminobenzidine (DAB, ZLI-9018, ZSGB-BIO) as a chromogen was used to visualize the reaction, which was finally stopped by removing the DAB and washing with running water.

### Hematoxylin and eosin staining.

The ovaries, uterus, and vagina of ZIKV-infected or control *Ifnar1^−/−^* mice were fixed in 4% paraformaldehyde overnight and embedded in paraffin blocks in an automated tissue processor (Leica, Germany). For the observation of histomorphology, 5-μm sections were prepared, and serial sections were mounted on slides and deparaffinized for 30 min at 60°C. After rehydration through a graded series of ethanol, the sections were stained with hematoxylin and eosin (HE). For follicle classification counts, the whole ovary was cut into serial 8-μm sections. The numbers of follicles in every fifth section of H&E-stained entire ovaries was counted in a double-blinded manner and then multiplied by a 5-fold correction factor. Follicles were classified into five stages: primordial, primary, secondary, mature, and atretic follicles. Only follicles that had an oocyte nucleus were scored. Primordial follicles featured an oocyte surrounded by a layer of squamous granulosa cells. Primary follicles were observed to have a single layer of cuboidal granulosa cells. If a liquid-filled cavity was observed in multiple layers of cuboidal and dense granulosa cells, the follicle was classified as a secondary follicle, and in contrast, mature follicles had fewer layers and a larger cavity. However, in atretic follicles, oocytes were aberrant, and/or multiple layers of granulosa cells were pycnotic and incompact (51).

### Papanicolaou staining.

**FIG 17:**
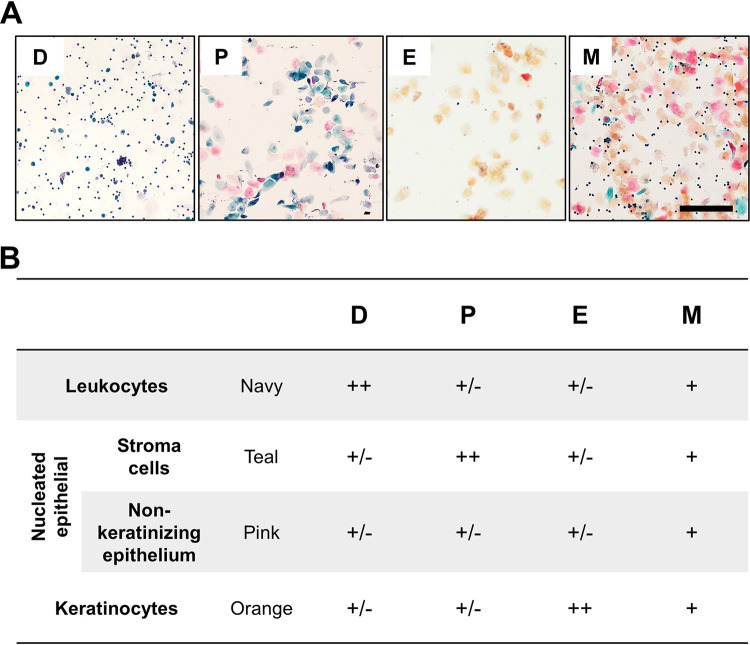
General criteria for determining the stage of the estrous cycle. Vaginal smears were collected from female mice and stained by the Papanicolaou method. The results were observed under a light microscope. D, diestrus; P, proestrus; E, estrus; M, metestrus. Bar = 200 μm. ++, large quantity; +, moderate quantity; +/−, dispensable quantity.

The estrous cycle stage was assessed by vaginal cytology. Female *Ifnar1^−/−^* mice were cohoused in groups, and vaginal smears were regularly collected for at least 7 days between 8 a.m. and 9 a.m. Exfoliated vaginal cells were collected by lavage with 50 μl sterile PBS. The lavage fluid was placed on dry glass slides. After air-drying naturally, the slides were first immersed in 95% ethanol for 4 min for fixation; then sequentially immersed in 85% ethanol, 80% ethanol, 70% ethanol, and purified water; and stained with hematoxylin. After differentiation by hydrochloric acid ethanol, the slides were sequentially stained with G-6 orange and EA-50, washed in 95% ethanol, and examined under a light microscope. The estrous cycle stages (estrus, metestrus, diestrus, and proestrus) were monitored on the basis of the cellular profile of each smear, as shown in the supplementary information (Fig. 17) (52).

### Western blot.

Ovaries were homogenized on ice in lysis buffer containing a complete protease and phosphatase inhibitor cocktail (Thermo Scientific) and centrifuged at 12,000 rpm for 15 min at 4°C. The amount of lysis buffer added to the centrifuge tube was proportional to the weight of the ovaries. The lysates (equal amount) were separated using 10% SDS-PAGE and transferred to polyvinylidene difluoride (PVDF) membranes (GE Healthcare). The membranes were blocked with TBS buffer containing 10% skim milk and incubated with primary antibodies against CYP19A1 (1:1,000, Abcam, ab18995), CYP17A1 (1:1,000, Abcam, ab125022), or β-actin (1:5,000, Cell Signaling Technology, 4970S) overnight at 4°C. The membranes were then incubated with a goat anti-rabbit secondary antibody (1:5,000, LI-COR, 926-68071), and an infrared laser imager (Odyssey CLX) was used to visualize the protein band.

### Quantification and statistical analysis.

Data visualization and statistical analyses were performed in GraphPad Prism 7.0. The normality of the data sets was determined by a Kolmogorov-Smirnov test. The data with normal distributions were analyzed by ordinary one-way analysis of variance (ANOVA) followed with *post hoc* tests, while those with abnormal distributions or heterogeneity of variance were analyzed by the nonparametric Mann-Whitney test or Kruskal-Wallis one-way ANOVA. The data are presented as the means ± standard deviation or means ± standard error of the mean (SEM). *P* < 0.05 (*) and *P* < 0.01 (**) were considered statistically significant and highly significant, respectively.
